# Phase dependent encapsulation and release profile of ZIF-based biocomposites†

**DOI:** 10.1039/c9sc05433b

**Published:** 2020-02-13

**Authors:** F. Carraro, M. de J. Velásquez-Hernández, E. Astria, W. Liang, L. Twight, C. Parise, M. Ge, Z. Huang, R. Ricco, X. Zou, L. Villanova, C. O. Kappe, C. Doonan, P. Falcaro

**Affiliations:** Institute of Physical and Theoretical Chemistry, Graz University of Technology Stremayrgasse 9 Graz 8010 Austria paolo.falcaro@tugraz.at; Department of Chemistry and the Centre for Advanced Nanomaterials, The University of Adelaide Adelaide South Australia 5005 Australia; Institute of Chemistry, University of Graz, NAWI Graz Heinrichstrasse 28 8010 Graz Austria; Dipartimento di Chimica Industriale “Toso Montanari”, Universita' di Bologna Viale del Risorgimento 4 Bologna Italy; Department of Materials and Environmental Chemistry, Stockholm University 106 91 Stockholm Sweden; Faculty of Technical Chemistry, Chemical and Process Engineering, Biotechnology, Graz University of Technology Petersgasse 10-12 8010 Graz Austria

## Abstract

Biocomposites composed of Zeolitic Imidazolate Frameworks (ZIFs) are generating significant interest due to their facile synthesis, and capacity to protect proteins from harsh environments. Here we systematically varied the composition (*i.e.* relative amounts of ligand (2-methylimidazole), metal precursor (Zn(OAc)_2_·2H_2_O), and protein) and post synthetic treatments (*i.e.* washes with water or water/ethanol) to prepare a series of protein@ZIF biocomposites. These data were used to construct two ternary phase diagrams that showed the synthesis conditions employed gave rise to five different phases including, for the first time, biocomposites based on ZIF-CO_3_-1. We examined the influence of the different phases on two properties relevant to drug delivery applications: encapsulation efficiency and release profile. The encapsulation efficiencies of bovine serum albumin and insulin were phase dependent and ranged from 75% to 100%. In addition, release profiles showed that 100% protein release varied between 40 and 300 minutes depending on the phase. This study provides a detailed compositional map for the targeted preparation of ZIF-based biocomposites of specific phases and a tool for the straightforward analysis of the crystalline phases of ZIF based materials (web application named “ZIF phase analysis”). These data will facilitate the progress of ZIF bio-composites in the fields of biomedicine and biotechnology.

## Abstract 2

We report two ternary phase diagrams that show the synthesis conditions to prepare protein@ZIF biocomposites with different phases, including BSA@ZIF-C and insulin@ZIF-C. For each biocomposite, we measured distinct encapsulation efficiency and release profile properties.

## Graphical abstract

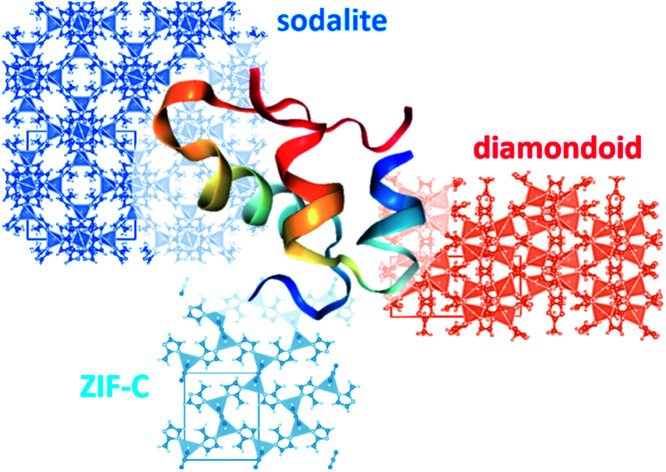

## Introduction

Metal–Organic Frameworks (MOFs) are a class of extended materials synthesized *via* a modular approach from inorganic (metal clusters or ions) and organic components that typically possess high surface areas and pore volumes.^1^ By carefully selecting the framework building units and reaction conditions, the chemistry, porosity and particle size of MOFs can be precisely controlled. These properties have attracted researchers to explore MOFs, and their composites, for a variety of applications including biomedicine.^2^ For example, MOF particles have shown unprecedented properties for the uptake and release of synthetic drugs,^3,4^ and more recently have been integrated with fragile biotherapeutics^5,6^ to improve their stability.^7,8^

Recently, Zeolitic Imidazolate Frameworks (ZIFs)^9,10^ were used to encapsulate biomacromolecules and to form bio-active composites.^11–16^ The most explored ZIF material for the encapsulation of bioentities is ZIF-8 which is composed of Zn^2+^ cations and 2-methylimidazole (HmIM). ZIF-8-based biocomposites form spontaneously in water around negatively charged biomacromolecules without any additives.^17^ This specific process has been termed *biomimetic mineralization* due to its broad similarities to naturally occurring biomineralization.^11,18,19^ The ZIF matrix has been shown to protect fragile biomacromolecules and assemblies thereof (*e.g.* viruses and living cells) from conditions that typically lead to loss of their activity and also act as a vector for *in vitro* and *in vivo* delivery.^11,12,15,19–23^ With respect to drug delivery applications, the biomimetic mineralization approach yields high encapsulation efficiencies (EE%) for biomacromolecules, typically ranging from 80% to 100%.^11,18,21^ In general, high EE% values are relevant to drug delivery applications as the therapeutic is the valuable component of the composite.^18^ Release of the biomolecules is achieved *via* decomposition of the ZIF-8 matrix at pH values < 6.5, in the presence of chelating agents (*e.g.* ethylenediaminetetraacetic acid, EDTA), or in specific buffer solutions (*e.g.* phosphate-buffered saline, PBS).^18,24,25^

ZIF-8 is a crystalline microporous material with sodalite (***sod***) topology that is synthesized by mixing aqueous solutions of HmIM and Zn^2+^.^26–28^ However, by varying the synthetic conditions, other topologies can be obtained (*e.g.* diamondoid (***dia***), katsenite (***kat***), ZIF-L).^29–33^ Similarly, for ZIF-based biocomposites, a variety of topologies can be accessed by modulating the reaction conditions.^19,34^ In these studies, a fixed amount of biomacromolecule was employed while the concentrations of the ZIF components were varied. Thus, the network topology was controlled by the relative amount of ligand and cation. In a subsequent study we observed that increasing the biomolecule concentration (*i.e.* carbohydrates) and maintaining a fixed HmIM: Zn^2+^ ratio also led to a change in topology from ***dia*** to ***sod***.^18^

This was most likely due to the dependence of sugar concentration on the pH of the reaction solution as the final solid ZIF product did not contain any carbohydrate. In addition to varying the relative concentration of the ZIF components and biomacromolecules, we have also found that post synthesis treatments (*e.g.* washing procedure) can trigger phase transitions.^18,34^

Though the various ZIF topologies share the same chemical connectivity, they can exhibit vastly different physical and chemical properties. For example, ZIF (***sod***) has an accessible porosity of *ca.* 1800 m^2^ g^−1^,^28^ while ZIF (***dia***) is non porous to N_2_.^32^ In addition, each topology possesses a distinct density and surface chemistry which influences their chemical stability.^35,36^ Accordingly, for biomedical applications, such as drug delivery, we posit that the precise control of topology is critical for the design of a carrier with specific release profiles. In this present work, for the first time, we systematically explored how the combination of the ratio of ZIF components, biomolecule concentration and washing procedure determines the structural phase of the biocomposite.

Here, we screened 36 compositions varying the weight fractions of HmIM, Zn(OAc)_2_·2H_2_O, and Bovine Serum Albumin (BSA). BSA was selected as a model biomacromolecule since it has been widely employed in the literature as a standard inexpensive protein for the preparation of biocomposites.^37–39^ The washing procedure was carried out using either water only or water and ethanol. The resulting solids were analysed by X-ray diffraction (XRD) and their topologies represented in ternary phase diagrams namely *TD-H*_*2*_*O* (water washed) and *TD-EtOH* (water and ethanol washed). A noteworthy result of this study is that we identified proteins encapsulated within ZIF-CO_3_-1, a ZIF previously obtained using solvothermal synthesis in absence of biomacromolecules.^40^ For each distinct phase: amorphous, U13, sodalite, diamondoid, and ZIF-CO_3_-1 (here referred as ***am***, ***U13***, ***sod***, ***dia*** and ***ZIF-C***, respectively) we selected and characterised a representative sample using scanning electron microscopy (SEM), vibrational spectroscopy (Fourier Transformed Infrared, FTIR, and Raman), and energy-dispersive X-ray spectroscopy (EDX).

Given the potential application of these biocomposites to drug delivery, for each identified phase we determined the encapsulation and release profiles of BSA and insulin (a clinical bio-therapeutic). Our results show that each biocomposite has high encapsulation efficiency EE% and a distinct release profile. These data will inform and facilitate future research in the burgeoning area of MOF-based drug delivery.

## Results and discussion

To explore the entire space of the variables, we selected compositions that were equally distributed within the phase diagram (see Fig. S1 in ESI†).

We prepared 36 different samples by varying the composition of zinc acetate, HmIM and BSA (each restricted to a mass fraction range of 10–80%, see Fig. S1 and Table S1 in ESI†) in a fixed volume of water (2 mL, see ESI† for Experimental details) and examined their topology *via* XRD.

**Fig. 1:**
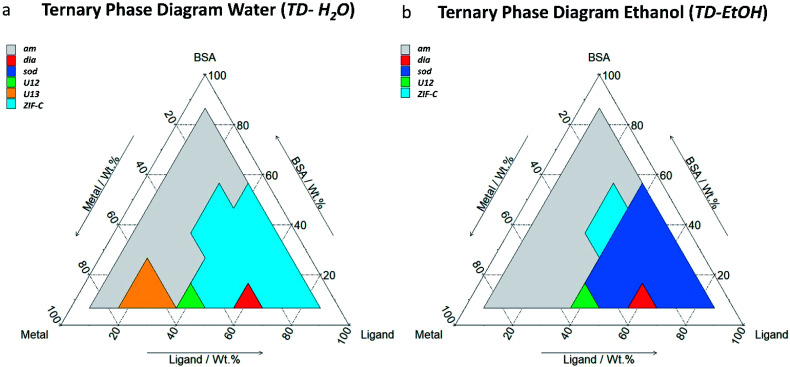
Ternary diagrams (by weight fraction, see Table S1† for details) of BSA, HmIM (labelled as ligand) and Zn(OAc)_2_·2H_2_O (labelled as metal). TD-H_2_O (a) represents the main phases (>50% wt, see Table S2† for details) obtained by washing the sample with DI water. TD-EtOH (b) represents the main phases (>50% wt, see Table S3† for details) obtained by washing the sample first with DI water and then with ethanol. The total mass of the reagent was chosen by selecting a value in between those previous reported in the literature (see ESI† for further details).^11,34^

After mixing, the different solutions were left to stand at room temperature for 24 h. From each of the 36 vials, the solid was separated *via* centrifugation and divided in two parts. One part was washed with deionised (DI) water only and the other was washed with water and ethanol. The samples were then air dried and investigated by X-ray diffraction (XRD). The resultant phases are reported in the ternary diagrams that relate each polymorph to the relative composition of Zn(OAc)_2_·2H_2_O, HmIM, and BSA in the synthesis solution. Fig. 1a and b show the ternary diagrams of the samples washed with DI water (TD-H_2_O) and with DI water and ethanol (TD-EtOH), respectively. Specific details related to the washing procedures and measurement conditions are reported in ESI.†

**Fig. 2:**
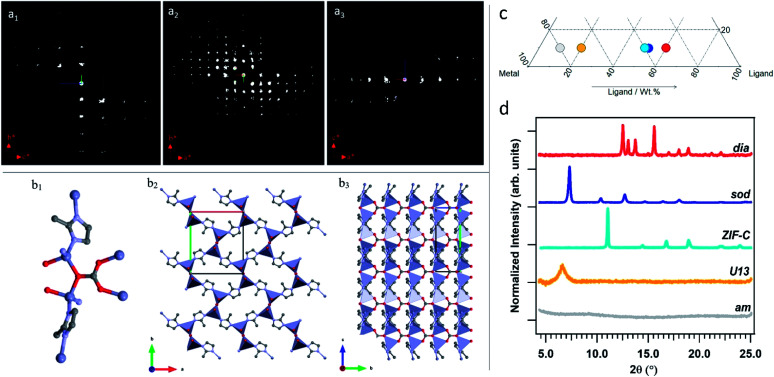
2D slice cuts from the reconstructed 3D reciprocal lattice show the 0*kl* (a_1_) *hk*0 (a_2_) and *h*0*l* (a_3_) planes. The scattering background (see Fig. S2†) was removed for clarification. Structure of ZIF-CO3-1: (b_1_) coordination mode of Zn; (b_2_) framework viewed along the c axis; (b_3_) framework structure viewed along the *a* axis. The Zn, O, N and C atoms are shown in blue, red, light blue and grey, respectively. (c) Section of the ternary diagram to highlight the samples selected as representative of the different phases (grey spot: amorphous biocomposite (TD-H_2_O); yellow spot: ***U13*** (TD-H_2_O); azure: ***ZIF-C*** (TD-H_2_O); blue: ***sod*** (TD-EtOH); red: ***dia*** (TD-EtOH)). The ***ZIF-C*** and ***sod*** samples were obtained with the same protocol, but with different washing procedures (TD-H_2_O and TD-EtOH, respectively). (d) XRD patterns of the amorphous biocomposite and of the biocomposites with ***dia***, ***sod***, ***ZIF-C*** topologies, and ***U13***.

When powders were washed only with DI water (TD-H_2_O) we observed the formation of an amorphous product (***am***) for small mass fractions of HmIM (10–20%). However, increasing HmIM to 20–30% while keeping BSA ≤ 20%, we found crystalline patterns dominated by a phase we have previously identified as ***U13***.^34^ Moving towards lower mass fractions of Zn^2+^ (10%), we measured amorphous diffraction patterns until BSA exceeded 50%. The remaining mass fraction ratios yielded diffraction patterns attributed to ***ZIF-C*** (Fig. 1a, S1 and Table S2 in ESI†). This was confirmed by continuous rotation electron diffraction (cRED, Fig. 2a, see ESI† for further details), which is a specialized technique for the structural determination of nanocrystals.^41,42^***ZIF-C*** is a high density framework (Fig. 2b) non porous to N_2_ (see Fig. S3, ESI†), that is prepared using solvothermal conditions (DMF/H_2_O, 140 °C)^40^ and not observed as a component of a biocomposites until this work. In a limited number of samples, ***ZIF-C*** was found mixed with previously reported patterns termed ***U12***, ***U13***, and ***dia*** topology.^34^

The TD-EtOH diagram indicates that ethanol washing gives rise to phase transitions: ***U13*** is no longer present and all the other samples with ***ZIF-C*** are partially or totally converted into ***sod***, with the exception of the ***dia***/***ZIF-C*** mixed phase (TD-H_2_O BSA/HmIM/Zn^2+^ = 10%/60%/30%) that transforms into ***dia***. Furthermore, ***U12*** is converted to a mixture of 3 phases (***U12***, ***sod***, ***ZIF-C***). Lastly, for all samples with mass fraction = 10% of Zn^2+^ and HmIM ≥ 40% we measured pure ***sod***. We observed diffraction patterns (*i.e.* crystalline) only for *ca.* wt_HmIM_ ≥ 30%, thus TD-EtOH confirms the important role of HmIM for the preparation of a crystalline material.

Combined, TD-H_2_O and TD-EtOH show the presence of 5 different phases (***am***, ***sod***, ***dia***, ***U13***, ***ZIF-C***) in their pure form or as compositions of phases (see Tables S2 and S3 ESI†). It is well known that the physical and chemical properties of ZIFs are dependent on their phase,^32,43,44^ thus we were motivated to examine the biomolecule encapsulation and release profiles of each biocomposite. However, first, we characterised each material by XRD, FTIR and Raman Spectroscopies, their elemental distribution *via* EDX and morphologies by SEM. In addition, given that biopharmaceuticals are the most expensive component of a drug delivery system^45–47^ we selected a biocomposite with a fixed 10 wt% of protein. Moving along this mass fraction we prepared the 5 different phases shown in Fig. 2c. The diffraction patterns plotted in Fig. 2d are univocally assigned to ***dia***, ***sod***, ***ZIF-C*** and ***U13*** (see Fig. S4, ESI†). For ***am*** the disordered state is confirmed by the absence of reflections. To facilitate the progress of Zn-mIM bio-composites towards biomedicine and biotechnology, we have developed a web application (https://rapps.tugraz.at/apps/porousbiotech/ZIFphaseanalysis/) named *ZIF phase analysis*. By uploading diffraction patterns collected using Cu Kα radiation, this web application allows for (1) a rapid identification of the crystalline phases, and (2) a rough estimation of the relative amounts (wt%). The web application was developed using the statistical software R – shiny package.^48^ Additional information can be found in ESI.†

**Fig. 3:**
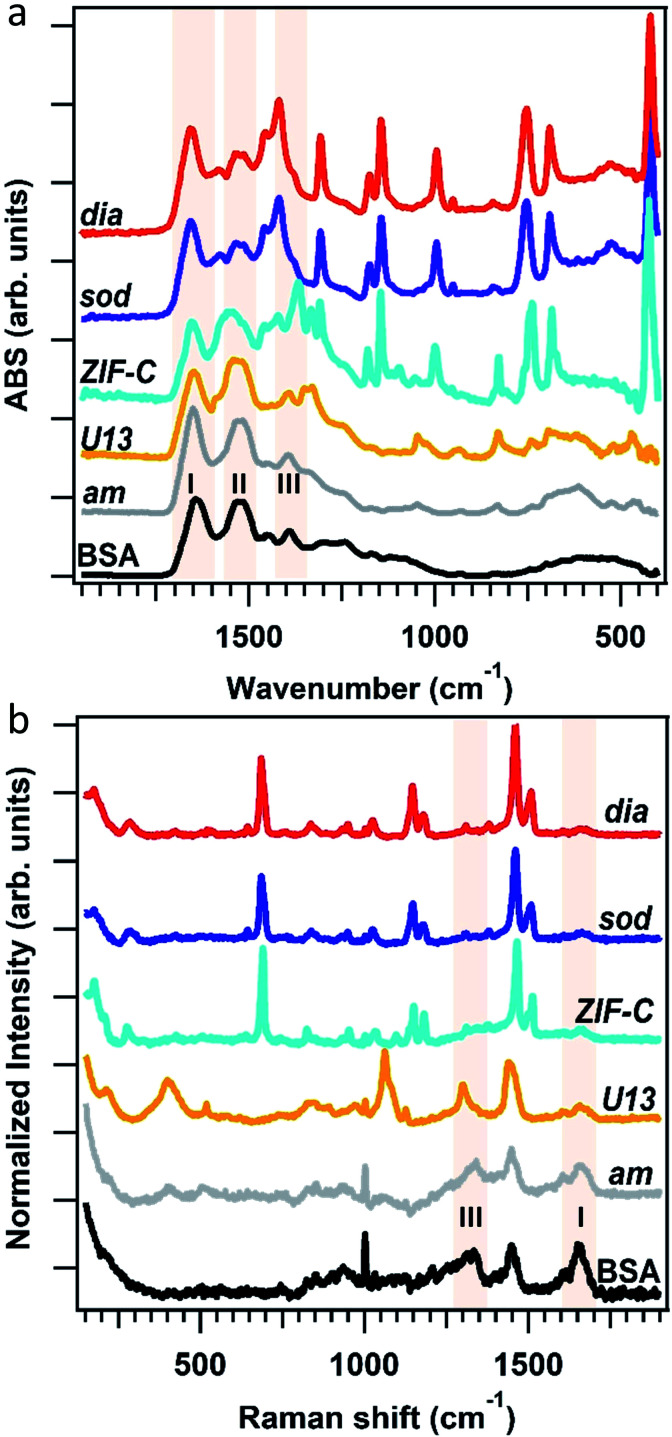
FTIR (a) and Raman (b) spectra of BSA, the biocomposites with ***am***, ***dia***, ***sod***, ***U13*** and ***ZIF-C*** phases. The spectral regions of Amide I, II and III bands of BSA are highlighted in light pink.

**Fig. 4:**
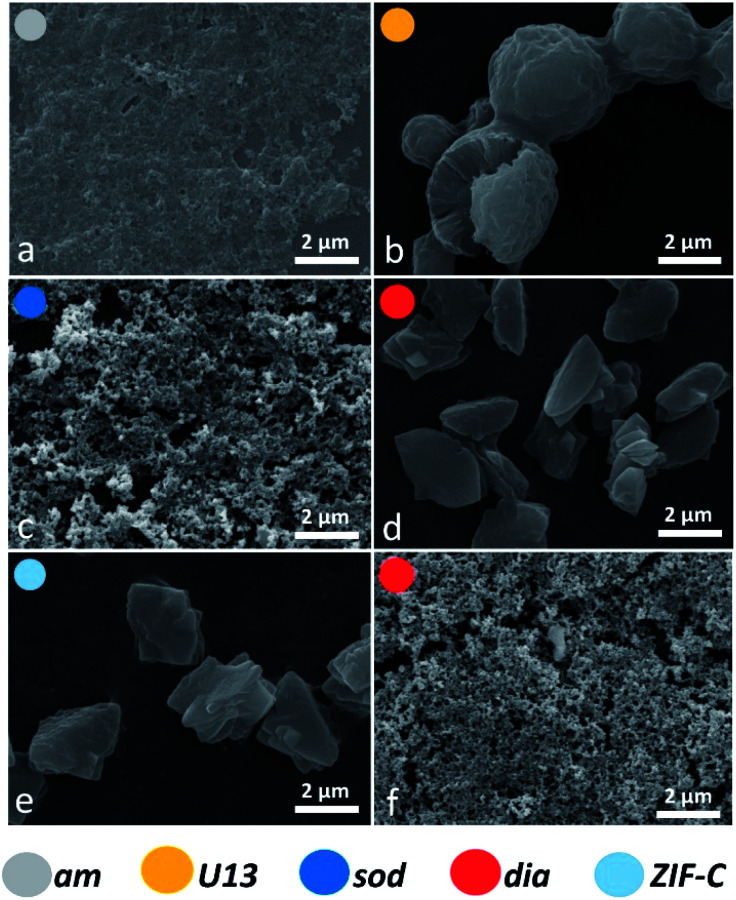
SEM micrographs of the biocomposites ***am*** (a) and ***U13*** (b, from TD-H_2_O), ***sod*** (c, from TD-EtOH), ***dia*** (from TD-H_2_O (d) and from TD-EtOH (f)) and ***ZIF-C*** (e, from TD-H_2_O) phases. ***ZIF-C*** refers to ZIF-CO_3_-1.^40^

To assess the connectivity and chemical composition of the bio-composites, we examined powder samples of ***am***, ***dia***, ***sod***, ***U13*** and ***ZIF-C*** using vibrational spectroscopy (Fig. 3). Analysis of the FTIR data confirms the presence of characteristic modes of the peptide backbone of BSA such as the amide I (1700–1610 cm^−1^) and amide II (1595–1480 cm^−1^) bands.^49,50^ The spectra of ***am*** and ***U13*** did not show vibrational modes that could be attributed to the imidazolate ligand. Furthermore, the vibrational mode at *ca.* 420 cm^−1^, assigned to the Zn–N stretching mode, is missing in the selected ***am*** and ***U13*** samples. This confirms that ***am*** and ***U13*** are not Zn(mIM)_2_-based polymorphs. Conversely, the spectra of ***sod***, ***dia*** and ***ZIF-C*** show several bands (420, 690, 752, 998, 1145, 1175, 1308, 1419, 1458, 1580 cm^−1^) typically observed for ***sod***-Zn(mIM)_2_.^31,34,51^ The spectrum of ***ZIF-C*** shows additional bands in the 700–850 and 1300–1400 cm^−1^ regions that can be assigned to bending and asymmetric stretching modes of CO_3_^2−^.^40^ Moreover, the Zn–N stretching mode is slightly shifted from 421 to 427 cm^−1^; we posit this is due to the different Zn-mIM coordination environment with respect to ***sod*** or ***dia*** topologies. The Raman spectra (160–1800 cm^−1^) of the same samples are reported in Fig. 3b. The ***sod*** and ***dia*** topologies show the typical Raman fingerprint of ***sod***.^52,53^ For both ***sod*** and ***dia***, the main bands are assigned to methyl bending (1459 cm^−1^), C5–N stretching (1147 cm^−1^), imidazole ring puckering (690 cm^−1^) and Zn–N stretching (178 and 278 cm^−1^).^52,53^ Comparing the Raman spectra of ***ZIF-C*** to ***sod***, small differences can be observed at 1466 cm^−1^ (assigned to imidazole ring puckering and to methyl bending) and 1097 cm^−1^ (assigned to CO_3_^2−^ stretching mode).^52–54^ These data support the different Zn-mIM coordination environment. In all the three crystalline Zn(mIM)_2_ phases (***sod***, ***dia***, ***ZIF-C***) we could confirm the presence of BSA (1550–1720 cm^−1^, amide I).^55^ For ***am*** and ***U13*** the vibrational modes of BSA dominate the spectra with broad bands assigned to amide I (1600–1700 cm^−1^), amide III (1300–1350 cm^−1^) and –CH deformation (1445 cm^−1^).^55^ The broad band at 400 cm^−1^ could be attributed to Zn–O stretching. This indicates potential for zinc protein interactions.^56^ The elemental composition of the biocomposites estimated by EDX shows a Zn content of *ca.* 5 wt% in the case of ***sod***. This value increases to *ca.* 15% for ***am*** and *ca.* 18% for ***U13*** (details can be found in ESI, Fig. S5 and Table S5†), suggesting that ***am*** and ***U13*** are mainly composed of Zn and BSA. We note that Zn^2+^ cations and BSA have been shown to form solid particles.^57,58^ Next, we investigated the morphology of the different phases by SEM (Fig. 4). For the water washed samples, very small particles of indistinguishable morphology were observed for ***am*** (Fig. 4a). While for ***U13*** the image shows spherical particles, and for ***dia***-Zn(mIM)_2_ and ***ZIF-C*** aggregates of plates 2–3 μm in size (Fig. 4b, d and e, respectively). For the ethanol washed sample, the particle size was reduced to less than 100 nm (Fig. 4c, f and S6, ESI†), with the exception of ***U13***. In this case, a similar particle morphology is observed despite the phase transition to ***am*** (Fig. S7, ESI†). We hypothesise that the particle size change is due to a combination of the crystalline network rearrangement and the collapse of the polycrystalline clusters induced by the different surface tension during the ethanol wash.^59,60^

**Fig. 5:**
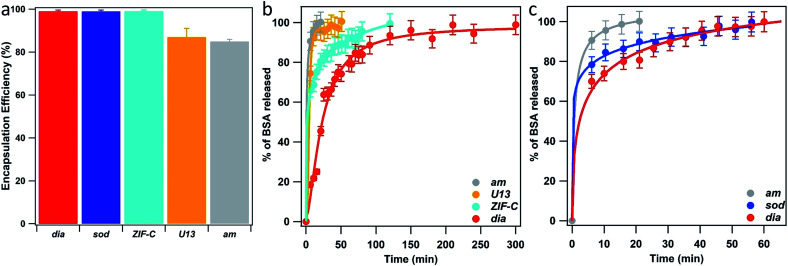
BSA encapsulation efficiency (EE%) (a) and BSA release profiles (from TD-H_2_O (b) and from TD-EtOH (c)) of the biocomposites with ***am***, ***U13***, ***sod***, ***dia*** and ***ZIF-C*** phases. ***ZIF-C*** refers to ZIF-CO_3_-1.^40^

We then turned our efforts to investigate the potential of using the 5 biocomposites ***am***, ***U13***, ***sod***, ***dia***, and ***ZIF-C*** as drug delivery systems. Initially BSA was employed as a model bio-therapeutic.^61,62^ For each sample a 10% mass fraction of BSA was employed in the synthesis (*vide supra*) and two important properties of a drug carrier were assessed: EE% and release profile.^63–65^ The estimated EE% (average of five independent analyses) for the different phases is shown in Fig. 5a. For each phase, high EE% values were observed (EE% > 85%) and, remarkably, ***dia***, ***sod*** and ***ZIF-C*** topology showed a 100% EE%. Details can be found in ESI†.

BSA release profiles were investigated to ascertain the quantity of protein released over time. As our study focuses on the examination of the crystalline phases and their properties rather than targeting a specific administration route, we examined release profiles at pH 5.5 as these conditions facilitate release of the cargo and simplifies a comparison between the different ZIF based particles. However, we note that different pH values will lead to different release profiles. The dissolution of each biocomposite was performed by exposing 1.08 mg of each phase to citric acid buffer solution (1 mL, 100 mM, pH 5.5, room temperature). We used UV-Vis and the Bradford Assay (see ESI† for details) to measure the amount of BSA in solution over time (see ESI† for details), and the release profiles are plotted in Fig. 5b and c. The experimental data points were fitted with a logistic fitting function^66^ which has previously been employed in the literature for the analysis of data related to the dissolution of different hydrophobic carries, including ZIF-8.^18,67,68^ Among the biocomposites washed only with water, ***am*** and ***U13*** showed the fastest dissolution: in 20 minutes, 100% of the encapsulated BSA was released (see ESI†) and the slowest release was measured for the ***dia*** topology: 100% of protein release was reached after 250 minutes. Whilst ***ZIF-C*** released 100% of the protein in 120 min. With respect to the ethanol-washed samples, ***am*** showed rapid release, *ca.* 20 min, and similar to the water washed samples, the slowest release was measured for the biocomposite of ***dia*** topology. However, in this case the release was significantly faster; 100% was observed in 60 minutes (compared to 250 min for the water washed samples). The different dissolution times observed for biocomposites of the same phase (*e.g.****dia***) can be attributed to particle size. However, when comparing the release profiles of topologically different particles the phase appears to play a dominant role. Thus, we can conclude that both the crystalline phase and the particle size play a crucial role in the design of MOF carriers for drug delivery applications.

**Fig. 6:**
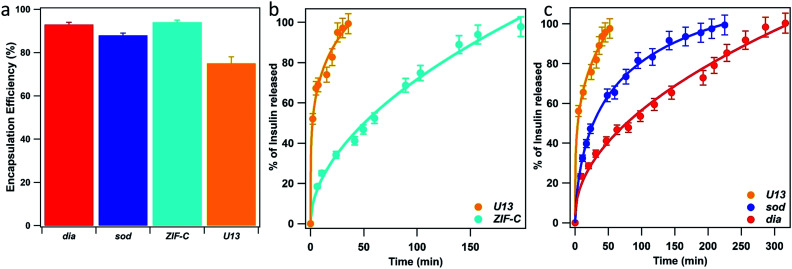
Insulin encapsulation efficiency (EE%) (a) and Insulin release profiles of the biocomposites with ***U13*** and ***ZIF-C*** phases (b, water washed samples) and ***U13***, ***sod*** and ***dia*** (c, EtOH washed samples) phases. ***ZIF-C*** refers to ZIF-CO_3_-1.^40^

To explore the potential biomedical applications of these biocomposites for the delivery of therapeutics we determined the release profiles of insulin encapsulated within the same 5 phases as studied for BSA. XRD data confirmed the expected phases (see Fig. S8, ESI†). However, for the ***dia*** phase an impurity of ***sod*** was present (15 wt%). The morphology of insulin biocomposites were investigated *via* SEM (Fig. S9 ESI†). The ***U13*** samples formed large micrometer sized star-like aggregates. Insulin@***ZIF-C*** is composed of both micrometer sized aggregates and small particles (<100 nm). We note the micrometer particles are of analogous morphology to the BSA@***ZIF-C*** particles. Similar to BSA@***dia***, insulin@***dia*** is composed of small particles (<100 nm). The insulin@***sod*** particles are composed of aggregates of small nanoparticles (100–200 nm). As expected, the insulin-based biocomposites (Fig. S10 ESI†) afford similar FTIR spectra to those of the BSA. For the samples with ***dia***, ***sod*** and ***ZIF-C*** topology we measured 93, 88 and 94% of insulin EE%, respectively (Fig. 6a). The lowest value was found for ***U13*** sample (EE% = 75%). Then, we tested the insulin release profiles (Fig. 6b and c). Among the crystalline water washed samples, the ***U13*** biocomposite showed the fastest release: in the first 40 minutes 100% of the encapsulated insulin was released. The slowest release was measured for the sample that possesses ***ZIF-C*** topology: the insulin release is 50% in 60 min and 100% in 200 min. Among the ethanol washed crystalline samples, ***U13*** showed the fastest release and a profile comparable to the water washed ***U13*** sample. The slowest release was measured for the sample that possesses ***dia*** topology: 100% of release was reached in 300 minutes. The sample that possesses ***sod*** topology showed an intermediate release profile (100% of insulin released in 220 minutes). In the context of insulin delivery systems, transdermal delivery is being explored as a less invasive method of administration.^69^ The measured cargo release times from ZIF-based biocomposites are similar to previously reported transdermal delivery systems.^70,71^ The difference in the release profiles from BSA and insulin suggests that the chemical nature, charge, and size of the protein could influence defects in the biocomposites.

To further validate the relevance of BSA ternary diagrams as a guide for the synthesis of other systems, we prepared a series of horseradish peroxidase (HRP) biocomposites. Analogous to BSA and insulin-based biocomposites, XRD data confirmed the presence of ***am***, ***U12***, ***ZIF-C*** and ***sod*** phases for the selected compositions (Fig. S11 ESI†). Although analogous synthetic conditions yielded to biocomposites with the same crystalline phases for BSA, insulin, and HRP, we hypothesise that the intrinsic heterogeneity of proteins (*e.g.* isoelectric point, hydrophobicity) could lead to deviations from the here proposed trend.

## Conclusion

We investigated the dependence of the crystal phases on the mass fraction of precursors (BSA or insulin, Zn(OAc)_2_·2H_2_O, HmIM) and the washing procedure (water or ethanol). For BSA we prepared 36 samples, washed only with water; the crystal phases were used to plot a ternary phase diagram (TD-H_2_O). More than 40% of the samples were found to be amorphous and the remaining samples where crystalline and non-porous (***dia***, ***U13***, ***ZIF-C***). Then, we tested the effect of ethanol washes on the 36 samples and found that it gave rise to phase transitions. For example, ***U13*** became amorphous, while ***ZIF-C*** transformed partially or completely into ***sod***. From these data we constructed a new second ternary phase diagram (TD-EtOH). Approximately 50% of the samples in TD-EtOH are amorphous; the remaining crystalline samples are dominated by the porous ***sod*** topology. The two ternary diagrams were used for the design of BSA-based composites with different crystallinity: starting with the same amount of protein, we could select conditions for the preparation of 5 different crystalline phases. To assess the potential of these systems for application to drug delivery, we focused our attention on determining their encapsulation efficiency and release profiles. We measured encapsulation efficiencies over 85% and a 100% release that can be tuned from 20 to 300 min depending on the selected phase. In general, we believe that the ternary diagrams can be used to design new biocomposites with tailored functional properties for bio-catalysis, bio-banking and drug delivery. As a proof of concept, we applied the ternary diagram to synthesize insulin biocomposites and test their encapsulation and release properties. Finally, we uncovered for the first-time proteins@***ZIF-C*** composites. For BSA@***ZIF-C*** and insulin@***ZIF-C***, we measured EE_BSA_% = 99% and EE_insulin_% = 94%, and 100% release was achieved in 120 and 200 min, respectively. This new bioMOF composite is an appealing crystalline structure alternative to ***sod*** and ***dia*** with potentially useful properties for encapsulation and release of biotherapeutics.

## Conflicts of interest

There are no conflicts to declare.

## Supplementary Material

SC-011-C9SC05433B-s001

SC-011-C9SC05433B-s002
