# Supplementary material for: Phase dependent encapsulation and release profile of ZIF-based biocomposites
Source: Chem Sci. 2020 Feb 13;11(13):3397–404. doi: 10.1039/c9sc05433b (PMC8529536; doi:10.1039/c9sc05433b)
Supplement: SC-011-C9SC05433B-s001 [file SC-011-C9SC05433B-s001.pdf]

Electronic Supplementary Material (ESI) for Chemical Science.
This journal is © The Royal Society of Chemistry 2020

Supporting Information

Phase dependent encapsulation and release profile of ZIF-based biocomposites

F. Carraro,[a] M. de J. Velasquez-Hernandez,[a] E. Astria,[a] W. Liang,[b] L. Twight,[a] C. Parise,[a,c,d] M. Ge,[e] Z. Huang,[e] R. Ricco,[a] X. Zou,[e] L. Villanova,[f] C. Oliver Kappe,[c] C. Doonan[b], P. Falcaro*[a,b]*

a) Institute of Physical and Theoretical Chemistry, Graz University of Technology, Stremayrgasse 9, Graz 8010, Austria
b) Department of Chemistry and the Centre for Advanced Nanomaterials, The University of Adelaide, Adelaide, South Australia 5005, Australia
c) Institute of Chemistry, University of Graz, NAWI Graz, Heinrichstrasse 28, 8010 Graz, Austria
d) Dipartimento di Chimica Industriale "Toso Montanari", Universita' di Bologna, Viale del Risorgimento 4, Bologna
e) Department of Materials and Environmental Chemistry, Stockholm University, 106 91 Stockholm, Sweden
f) Faculty of Technical Chemistry, Chemical and Process Engineering, Biotechnology, Graz University of Technology, Petersgasse 10-12, 8010 Graz, Austria

Contents:

Experimental section. Pages 3-5

Table S1: List of the synthesize samples for each Ternary Diagram, together with the details about the weight percentages of the different components and the ligand/metal weight and molar ratios in the starting solution mixture. Page 6.

Table S2: List of the weight % of the different phases calculated from the diffraction patterns of the samples of the TD-H2O. The data were analyzed using the “ZIF phase analysis“ application. Page 7.

Table S3: List of the weight % of the different phases calculated from the diffraction patterns of the samples of the TD-EtOH. The data were analyzed using the “ZIF phase analysis“ application. Page 8.

Figure S1: Ternary diagrams (TD, by weight fraction) of BSA, HmIM (labelled as Ligand) and $Zn(OAc)_2{\cdot}2(H_2O)$ (labelled as Metal) with the 36 investigated points. Page 9.

Figure S2: The Reconstructed 3D reciprocal lattice from the cRED data. Page 9.

Table S4: Experimental parameters for cRED data collection and crystallographic data. Page 10

Figure S3: N2 77K isotherms of the ZIF-C biocomposite (Sample #29 of Table S1), the sod biocomposite obtained by washing with ethanol the ZIF-C sample and the sod biocomposite treated at 325°C for 2h. Page 10.

Figure S4: XRD patterns of dia, sod and ZIF-C biocomposites and of the calculated pure MOFs. Page 11.

Figure S5: EDX spectra of patterns of BSA, of the amorphous biocomposite and of the biocomposites with dia, sod, ZIF-C, and U13 topology. Page 11.

Table S5: Atomic % calculated from EXD spectra. Page 12.

Figure S6: SEM micrographs of the dia (left) and sod(right) samples obtained by washing the samples with ethanol (TD-EtOH). Page 12.

Figure S7: SEM micrograph of the am sample obtained by washing with ethanol the U13 sample. Page 13.

Figure S8: XRD patterns of the different phases of the Insulin biocomposites. Page 13

The “ZIF phase analysis“ application – Version 1.0.0 Pages 14-23.

Figure S9. Example of accepted data file of the “ZIF phase analysis“ application – Version 1.0.0. Page 14.

Table S6. 2θ values and RIR factors of selected peaks used for the identification of five ZIF phases, biocomposites with U12 and U13 structure and two references (ZnO and ZrO_2). Page 23.

Experimental section

Synthesis

In a typical experiment for ZIF polymorph synthesis, 1 mL of an aqueous solution of $Zn(OAc)_2 \cdot 2(H_2O)$ (EMSURE, Merck) was added to a 1 mL of an aqueous solution of HmIM (TCI Chemicals) and Bovine Serum Albumin (BSA, lyophilized powder, Sigma-Aldrich). The total volume of each synthesis was 2 mL. Deionized (DI) water was used for all the experiments. The relative weight percentage of the three components ($Zn(OAc)_2 \cdot 2(H_2O)$, BSA, HmIM) were systematically varied from 10 to 80 wt%, starting from the following aqueous stock solutions: 80 mM $Zn(OAc)_2 \cdot 2(H_2O)$, 440 mM HmIM and 36 mg/mL of BSA. For all the investigated samples, the total mass of the reagents is 43.8 mg (21.9 mg/ml). This value was chosen selcting a value in between the those of previous reported in the literaure for the shytnesis via the biomimetic mineralization method of BSA@ZIF-8 biocomposites.[i,ii] The detailed composition of each investigated point of the ternary diagram is summarized in Table S1. The reaction mixture was left under static conditions at RT for 24 h. Each sample was synthesized in a 2 mL Eppendorf Tube. After 24 hours the solid product was separated via centrifugation (13000 rpm for 5 min; centrifuge used: Eppendorf 5425) and the supernatant was discarded. Depending on the phase diagram (TD-H_2O, or TD-EtOH), the obtained powder pellet was then washed using one of two different protocols (see also Washing_Procedure.mp4):

TD-H_2O - Water washed materials:

The pellet was re-suspended in deionized water (1.5 mL) using a vortex mixer (3000 rpm for 1 minute, VELP Scientifica ZX3). The suspension was centrifuged (13000 rpm for 5 min) to yield a pellet and the supernatant was discarded. This washing procedure was repeated 6 times. Finally, the recovered powders were air-dried for 48 h at 25 °C.

TD-EtOH - Water and Ethanol washed materials:

The pellet was re-suspended in deionized water (1.5 mL) using a vortex mixer (3000 rpm for 1 minute, VELP Scientifica ZX3 The suspension was centrifuged (13000 rpm for 5 min) to yield a pellet (13000 rpm for 5 min) and the supernatant was discarded. This deionized water washing procedure was repeated 3 times. Then, the pellet was re-suspended in ethanol (1.5 mL) using a vortex mixer (3000 rpm for 1 minute, VELP Scientifica ZX3). The suspension was centrifuged (13000 rpm for 5 min) to yield a pellet and the supernatant was discarded. This ethanol washing procedure was repeated 3 times. Finally, the recovered powders were air-dried for 48 h at 25°C.

The mass percentages of the different phases calculated from the diffraction patterns of each investigated sample are summarized in Table S2 and S3 and in Figure S1.

Characterization

XRD

XRD patterns were acquired using a Rigaku SmartLab II equipped with a Cu anode (λ=1.5406 Å) and operating at 9 kW.

SEM-EDX

SEM micrographs and EDX spectra were collected using Tescan VEGA 3 SEM with tungsten source filament working at 20 kV. Prior the analysis the powder samples were dropcasted on a piece of Si (100) and sputter-coated with Gold.

ATR

FT-IR spectra were recorded on a Bruker ALPHA spectrometer using the ATR accessory with a diamond window in the range 400 – 4000 cm^{-1}.

RAMAN

Raman spectra were recorded on a Thermo Fisher DXR2 Microscope equipped with a 785 nm laser operating at 35 mW and a 10x objective.

Gas Sorption

Gas adsorption isotherm measurements were performed on an ASAP 2020 Surface Area and Pore Size Analyser. Samples were activated by heating in vacuum at 120 °C for 12 hours. UHP grade (99.999%) N_2 and He were used for all measurements. The temperatures were maintained at 77 K (liquid nitrogen bath).

Evaluation of Encapsulation Efficiency

Encapsulation Efficiency measurement from supernatant: the samples after synthesis (24 hours) were centrifuged 5 minutes 13000 rpm. The supernatant (1 mL) was recovered by micropipette. 50 µL of supernatant was mixed with 1500 µL of Bradford solution (Sigma-Aldrich, sample-to-Bradford ratio 1:30). The solution was left for 5 minutes at room temperature. Afterwards, this mixture was analyzed by UV-VIS (595 nm). All the experiments were performed in triplicates.

Encapsulation Efficiency measurement from destroyed MOFs: samples were washed 3X with water and 3X with ethanol (TD-EtOH) or 6X with water (TD-H_2O). The MOF was destroyed under acidic conditions using Citrate Buffer (2 mL, 100 mM pH 5.5). Then, 50 µL of the resultant clear solution was mixed with 1500 µL of Bradford solution (Sigma-Aldrich, sample-to-Bradford ratio 1:30). The solution was left for 5 minutes at room temperature. Afterwards, this mixture was analyzed by UV-VIS (595 nm). All the experiments were performed in triplicates.

This overall amount of BSA part of the biocomposite was then compared to the amount of BSA obtained by the dissolution (0.1 M citric acid aqueous solution at pH 5.5) of the washed biocomposites. This was useful for the evaluation of the effect of the washing procedure on the surface-adsorbed protein. Only in the case of ***am*** (72%) and ***U13*** (76%) we noticed a significant difference (>5%) in the calculated EE%. These results excluded release of a significant amount of protein from the ***sod***, ***dia***, ZIF-C bicomposites particles once exposed to ethanol.

Evaluation of BSA-release profile

Release test was performed using cumulative release method. The samples were mixed with 1 mL Citrate Buffer (100 mM pH = 5.5, room temperature). The samples were shaken using an orbital mixer. At regular intervals, the mixture was vortexed for 3 s and centrifuged 1 min. Then, 50 µL of the supernatant was taken and replaced with the same volume of fresh Citrate buffer. Then, the 50 µL of the supernatant was mixed with 1500 µL of Bradford solution (Sigma-Aldrich, sample-to-Bradford ratio 1:30). The solution was

left for 5 minutes at room temperature. Afterwards, this mixture was analyzed by UV-VIS (595 nm). All the experiments were performed in triplicates.

Transmission electron microscopic (TEM) analysis.

Samples for transmission electron microscopy observation were dispersed in deionized water. A droplet of the suspension was transferred onto a carbon-coated copper grid for each sample. Observation was performed on a JEOL JEM2100 microscope, and operated at 200 kV (Cs 1.0 mm, point resolution 0.23 nm). Images were recorded with a Gatan Orius 833 CCD camera (resolution 2048 x 2048 pixels, pixel size 7.4 µm) under low dose conditions. Electron diffraction patterns were recorded with a Timepix pixel detector QTPX-262k (512 x 512 pixels, pixel size 55 µm, Amsterdam Sci. Ins.).

Continuous Rotation electron diffraction (cRED) collection.

The data were collected using the software Instamatic1-3. A single-tilt holder was used for the data collection, which could tilt from -60° to +60° in the TEM. The area used for cRED data collection was about 1.0 µm in diameter. The speed of goniometer tilt was 0.45° s-1. The exposure time was 0.5 s per frame. Data was collected at room temperature within 4 min. The covered tilt angle was 93.91°.

As shown in the Inset of Figure S2, the size of the crystal is in the range of nanometers. Due to the tiny crystal size, the structural determination was conducted using cRED data. Figure S2 shows the reconstructed 3D reciprocal lattices from the cRED data. Unit cell parameters were determined to be a = 10.3 Å, b = 12.5 Å, c = 4.7 Å, α = 88.9°, β = 89.6°, and γ = 89.6°. As the lattice parameters α, β and γ are near 90°, it indicates that the possible crystal system could be othorhombic. From 3D projections and two-dimensional (2D) slice cuts of the 3D reciprocal lattice at 0kl, hk0 and h0l planes (Figure 2a, main text), the reflection conditions are 0kl: k=2n, h0l: h=2n, h00: h=2n, and 0k0: k=2n. Thus, the possible space groups are Pba2 (No. 32), and Pbam (No. 55). The space group Pba2 was chosen for further structural determination. These results are summarized in Table S4.

We investigated also the ***U13*** sample with cRED. However, in case of ***U13***, the limited crystallinity of the sample (only 1 broad peak at 6.6 °) does not allow for the identification of the unit cell.

Table S1: List of the synthesize samples for each Ternary Diagram, together with the details about the weight percentages of the different components and the ligand/metal weight and molar ratios in the starting solution mixture.

**SAMPLE #**	**Weight Percentage (%)**			**Ligand/Metal**	**Ligand/Metal**
	**$Zn(OAc)_2 \cdot 2(H_2O)$**	**HmIM**	**BSA**	**weight ratio**	**molar ratio**
1	10	10	80	1.00	2.70
2	20	10	70	0.50	1.35
3	30	10	60	0.33	0.90
4	40	10	50	0.25	0.68
5	50	10	40	0.20	0.54
6	60	10	30	0.17	0.45
7	70	10	20	0.14	0.39
8	10	20	70	2.00	5.30
9	20	20	60	1.00	2.65
10	30	20	50	0.67	1.77
11	40	20	40	0.50	1.33
12	50	20	30	0.40	1.06
13	60	20	20	0.33	0.88
14	70	20	10	0.29	0.76
15	10	30	60	3.00	8.00
16	20	30	50	1.50	4.00
17	30	30	40	1.00	2.67
18	40	30	30	0.75	2.00
19	50	30	20	0.60	1.60
20	60	30	10	0.50	1.33
21	10	40	50	4.00	10.70
22	20	40	40	2.00	5.35
23	30	40	30	1.33	3.57
24	40	40	20	1.00	2.68
25	50	40	10	0.80	2.14
26	10	50	40	5.00	13.30
27	20	50	30	2.50	6.65
28	30	50	20	1.67	4.43
29	40	50	10	1.25	3.33
30	10	60	30	6.00	16.00
31	20	60	20	3.00	8.00
32	30	60	10	2.00	5.33
33	10	70	20	7.00	18.70
34	20	70	10	3.50	9.35
35	10	80	10	8.00	21.30
36	80	10	10	0.13	0.34

Table S2: List of the weight % of the different phases calculated from the diffraction patterns of the samples of the TD-H_2O. The data were analyzed using the "ZIF phase analysis" application. The rows highlighted in gray represent amorphous samples.

TD-H_20	Weight Percentage (%)				
**SAMPLE #**	**sod**	**dia**	**ZIF-C**	**U13**	**U12**
1					
2					
3					
4					
5					
6					
7					
8					
9					
10					
11					
12					
13				100	
14				100	
15					
16			100		
17			100		
18					
19					
20				100	
21			100		
22			100		
23			100		
24			100		
25			40		60
26			100		
27			100		
28			100		
29			100		
30			100		
31			100		
32		89	11		
33			100		
34			100		
35			100		
36					

Table S3: List of the weight % of the different phases calculated from the diffraction patterns of the samples of the TD-EtOH. The data were analyzed using the "ZIF phase analysis" application. The rows highlighted in gray represent amorphous samples.

TD-EtOH	Weight Percentage (%)				
SAMPLE #	sod	dia	ZIF-C	U13	U12
1					
2					
3					
4					
5					
6					
7					
8					
9					
10					
11					
12					
13					
14					
15					
16	31		69		
17	5		95		
18					
19					
20					
21	59		41		
22	100				
23	97		3		
24	100				
25	28		21		51
26	100				
27	87		11		
28	92		8		
29	100				
30	100				
31	100				
32		100			
33	100				
34	100				
35	100				
36					

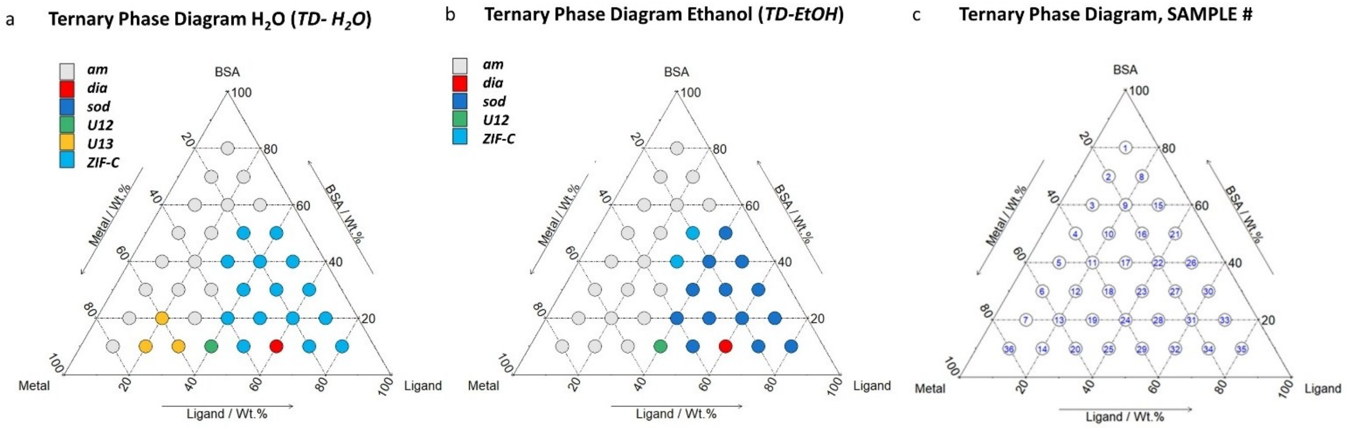

Figure S1: Ternary diagrams (TD, by weight fraction) of BSA, HmIM (labelled as Ligand) and $Zn(OAc)_2{\cdot}2(H_2O)$ (labelled as Metal) with the 36 investigated points. The colour of the points of TD-H_2O (a) represents the main phases obtained by washing the sample with DI water. The colour of the points of TD-EtOH (b) represents the main phases obtained by washing the sample first with DI water and then with ethanol. The TD in (c) highlights the sample # reported in Table S1, S2 and S3. For further details, see Tables S1, S2 and S3.

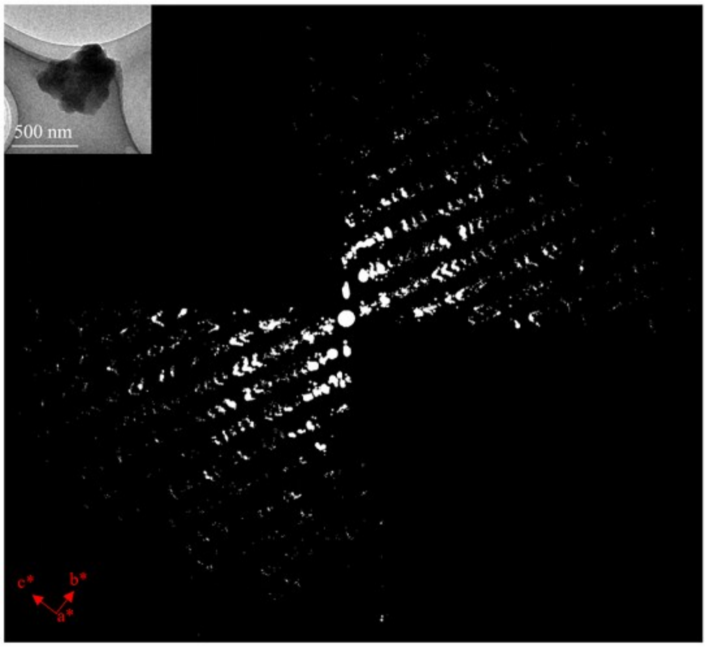

Figure S2: The Reconstructed 3D reciprocal lattice from the cRED data. The TEM micrograph of the investigated crystal is shown as an inset.

Table S4: Experimental parameters for cRED data collection and crystallographic data.

Tilt range (°)	-53.60° to 40.31°
Tilt rate (°/s)	0.45
Chemical formula	$C_9H_{10}N_4O_3Zn_2$
Z	2
Exposure time/frame (s)	0.5
Total number of frames	365
Data collection time (min)	3.46
Completeness (%)	0.766

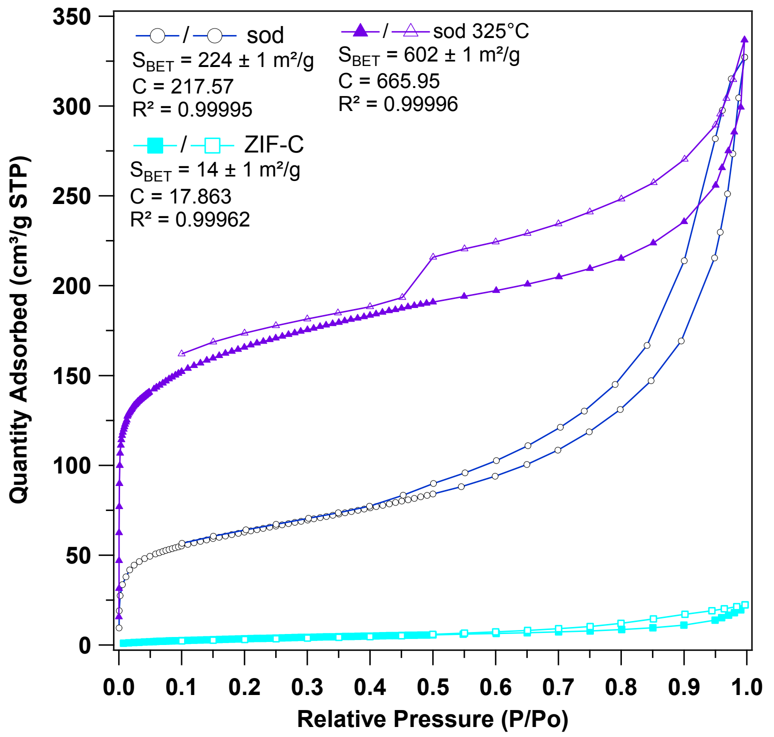

*Figure S3: N2 77K isotherms of the ZIF-C biocomposite (Sample #29 of Table S1), the sod biocomposite obtained by washing with ethanol the ZIF-C sample and the sod biocomposite treated at 325°C for 2h. To confirm that **ZIF-C** obtained in presence of BSA, we characterized this sample with nitrogen adsorption and desorption experiments at 77 K (Fig SX, ESI†). The calculated BET surface area was 14 m^2/g, confirming that **ZIF-C** is a non-porous material, similarly to **U12**, **U13** and **dia**-Zn(mIM)$_2$.[i] Motivated by the phase transition triggered by ethanol washes, the sample originally prepared as ZIF-C and then transformed into sod was investigated with the same N_2 physisorption setup. The N_2 sorption profiles and the calculated BET surface areas confirmed the presence of permanent microporosity (BET surface area: 224 m^2/g). We exposed the sample to thermal treatment to decompose the encapsulated BSA (325°C, 2h). In this case, the measured surface area was raised to 602 m^2/g indicating the gravimetric contribution of the protein prior thermal decomposition.*

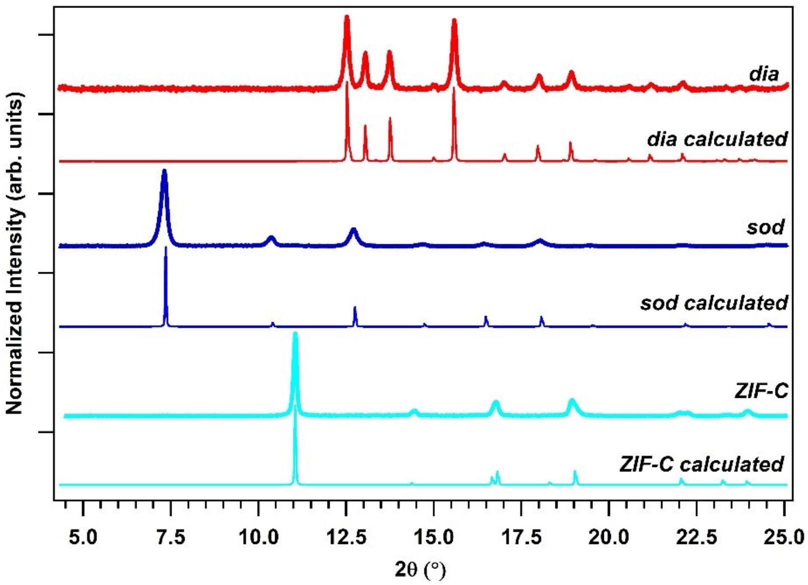

Figure S4: XRD patterns of dia, sod and ZIF-C biocomposites and of the calculated pure MOFs.

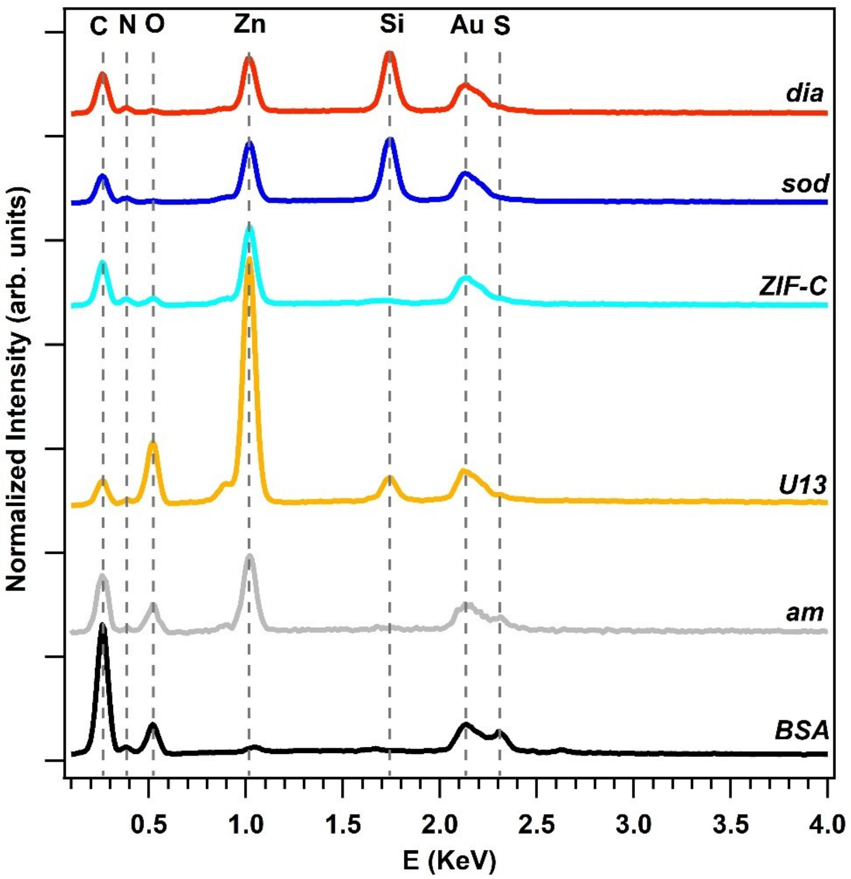

Figure S5: EDX spectra of patterns of BSA, of the amorphous biocomposite and of the biocomposites with dia, sod, ZIF-C, and U13 topology.

Table S5: Atomic % calculated from EXD spectra. The contribution from Au (coating) and Si (substrate) was not considered. For BSA, the remaining 2% was due to Na, Cl and S.

	BSA	am	U13	U15	sod	dia
**C**	61%	53%	40%	59%	66%	64%
**N**	16%	9%	6%	25%	24%	27%
**Zn**	0%	15%	18%	6%	5%	5%
**O**	21%	23%	36%	10%	4%	5%

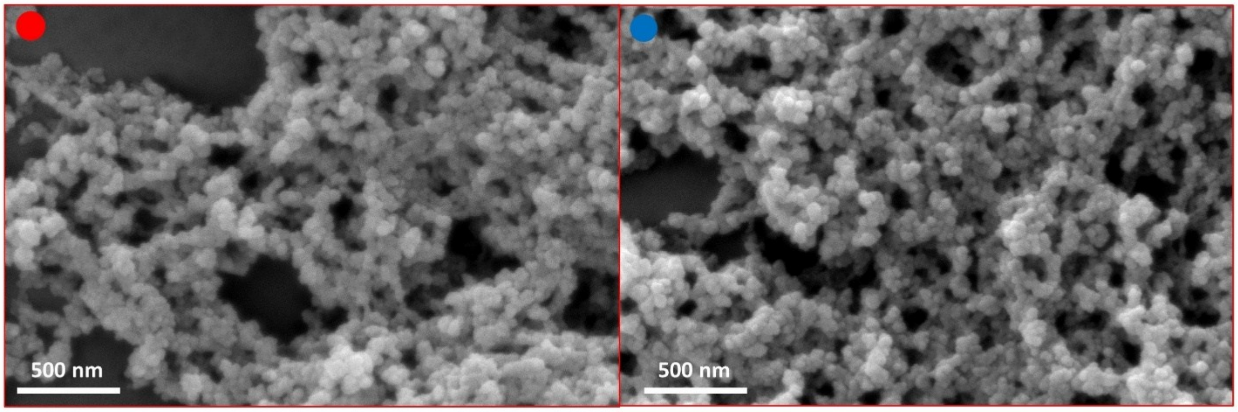

Figure S6: SEM micrographs of the dia (left) and sod(right) samples obtained by washing the samples with ethanol (TD-EtOH).

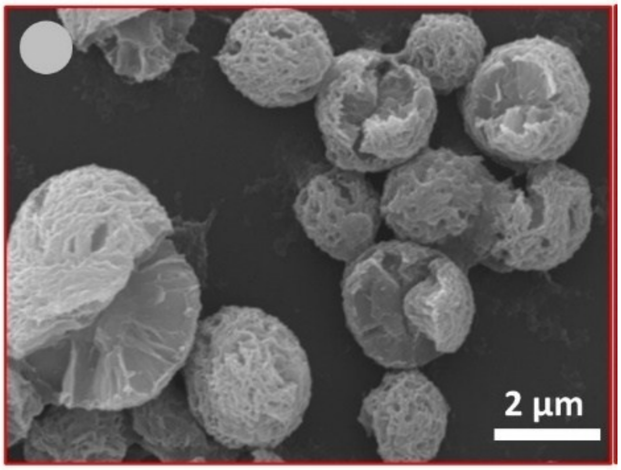

Figure S7: SEM micrograph of the am sample obtained by washing with ethanol the U13 sample.

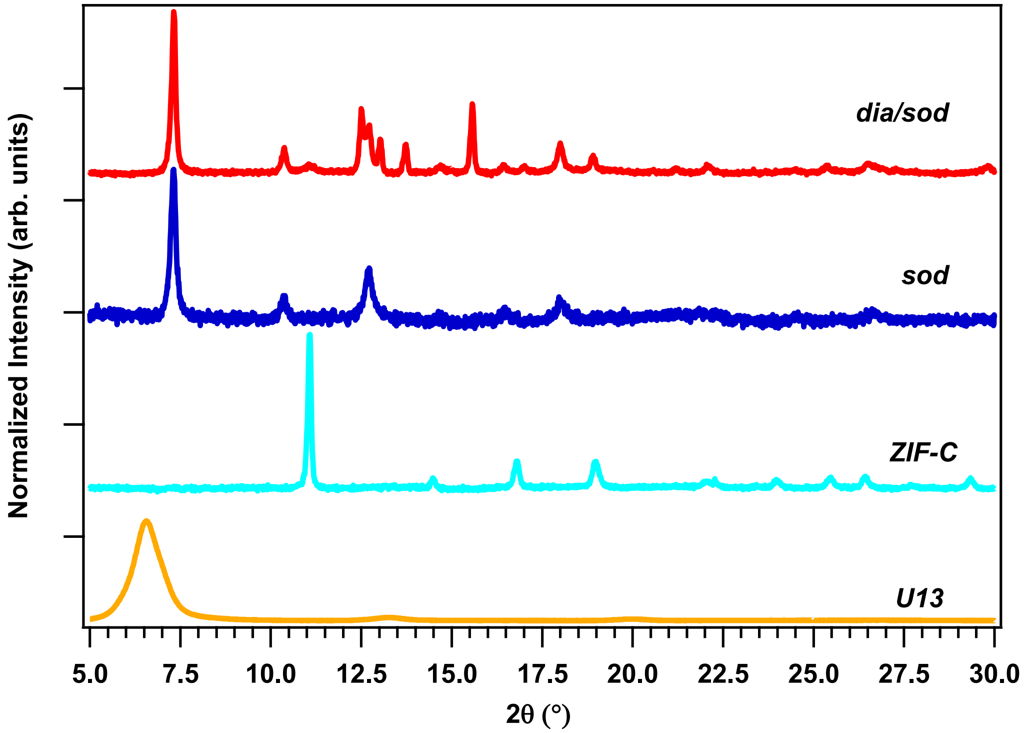

Figure S8: XRD patterns of the different phases of the Insulin biocomposites.

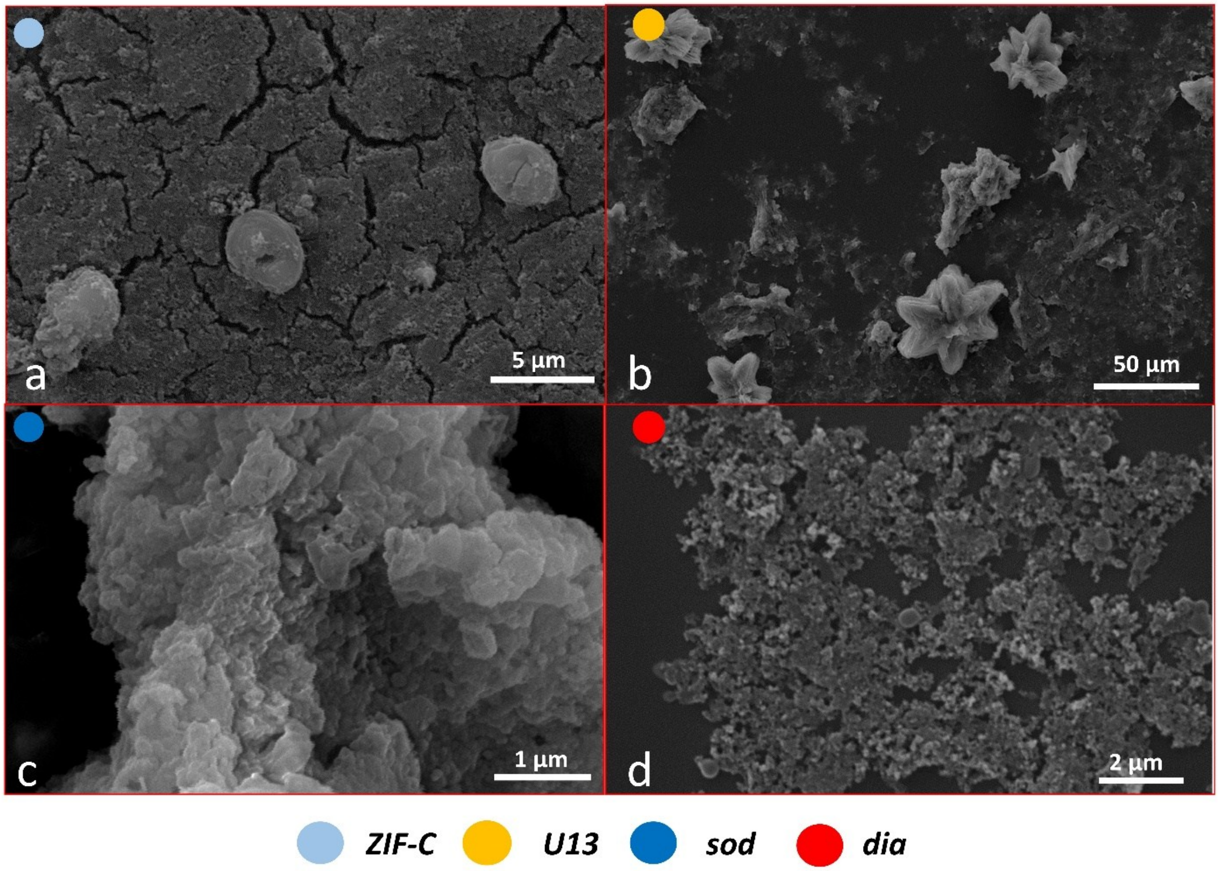

Figure S9: SEM micrographs of the insulin biocomposites with ZIF-C (a, from TD-H2O), U13 (b, from TD-H2O), sod (c, from TD-EtOH) and dia (d, from TD-EtOH) phases.

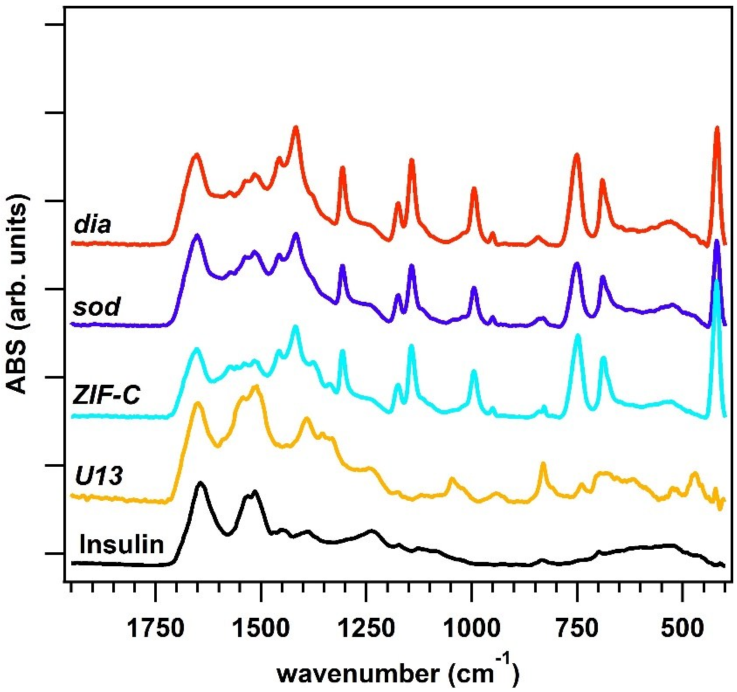

Figure S10: FTIR spectra of insulin and of the insulin-based biocomposites with dia, sod, U13 and ZIF-C phases. The analysis of the FTIR data confirms the presence of characteristic modes of the peptide backbone of insulin such as the Amide I (1700-1610 cm^{-1}) and Amide II (1595-1480 cm^{-1}) bands in all the examined polymorphs. As in the case of BSA@U13, the spectrum of insulin@U13 did not show vibrational modes that could be attributed to the imidazolate ligand and to the Zn–N bond. For insulin encapsulated in sod, dia and ZIF-C we detected several vibrational modes (420, 690, 752, 998, 1145, 1175, 1308, 1419, 1458, 1580 cm^{-1}) typically observed for sod-Zn(mIM)$_2$. Moreover, is possible to identify the ZIF-C additional bands in the 700-850 and 1300-1400 cm^{-1} regions, assigned to weak bending and asymmetric stretching modes of CO_3^{2-}.

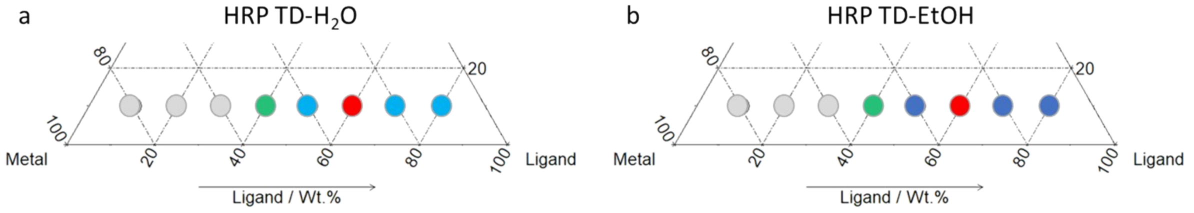

Figure S11: Investigated section of the HRP-based biocomposites ternary diagram (HRP wt%= 10%) of water (a) or ethanol (b) washed samples highlighting the obtained crystal phases (grey spot: amorphous biocomposite; azure: ZIF-C; blue: sod; red: dia; green: U12). We prepared the samples according to the protocols used to prepare the BSA biocomposites with an initial wt% of protein of 10%. According to the XRD results, we obtained am, U12, ZIF-C and sod biocomposites. U12 is obtained always in combination with ZIF-C (for the water washed samples) or ZIF-C and sod (for the ethanol washed samples) In general, the HRP samples followed the results obtained for BSA samples.

The “ZIF phase analysis“ application – Version 1.0.0

“ZIF phase analysis“ is an interactive web application built using R[iii] 3.5.3, RStudio[iv] 1.1.463 and the packages *shiny*[v], *shinydashboard*[vi], and *shinyalert*[vii]. The application is hosted at the Technical University of Graz and deployed on-premises using Shiny-Server.

The application is open worldwide and can be accessed with no restrictions via the URL https://rapps.tugraz.at/apps/porousbiotech/ZIFphaseanalysis/. A user can upload his/her own data file consisting of the diffraction pattern obtained via X-ray measurement of powders. The application processes the uploaded data file and returns the ZIF phase(s) identified in the investigated diffraction pattern.

The “ZIF phase analysis“ application comprises of the “Data“ tab and the “Analysis“ tab, both listed in the black sidebar on the left of the screen. The “Data“ tab is used to upload the data, whereas the “Analysis“ tab is used to perform the analysis and present the results.

The following sections provide details about i) accepted data file format, ii) use of the application for data upload and analysis, and iii) underlying statistical analysis.

Accepted data file format

The data file must consist of a first column indicating the angle 2θ in degrees (°) and a second column indicating the intensity (e.g. photon counts or photons/second). Columns headers are accepted; however, any other line of text must be removed from the data file. Various file formats (e.g. .txt, .dat) and column/decimal separators are accepted; additional details are reported in the section “Data file upload“. An example of accepted data file format is shown in **Figure S10**.

```
32W - Editor
Datei   Bearbeiten   Format   Ansicht   Hilfe

2.000 3532
2.013 3556
2.026 3401
2.040 3360
2.053 3234
2.066 3197
2.079 3176
2.093 3244
2.106 3153
2.119 3039
2.132 3078
2.146 2990
2.159 2956
```

Figure S12. Example of accepted data file. The file is named “32W.dat“. It has no header (i.e. column title) and comprises of two columns of numeric values indicating 2ϑ angles (first column) at which the intensity (second column) is measured. The columns are separated by a white space (column separator). A point decimal separator is used.

Data file upload

To visualize the content of the "Data" tab, click on "Data" in the sidebar (top left of screen).

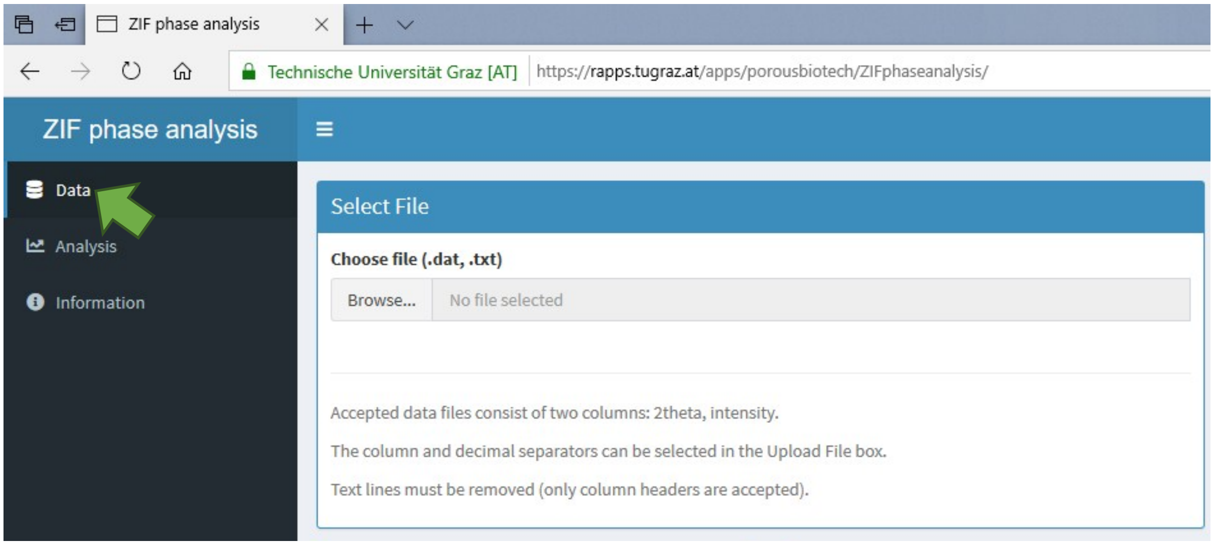

In the "Select File" box click on the "Browse" bottom and choose the data file to be analysed.

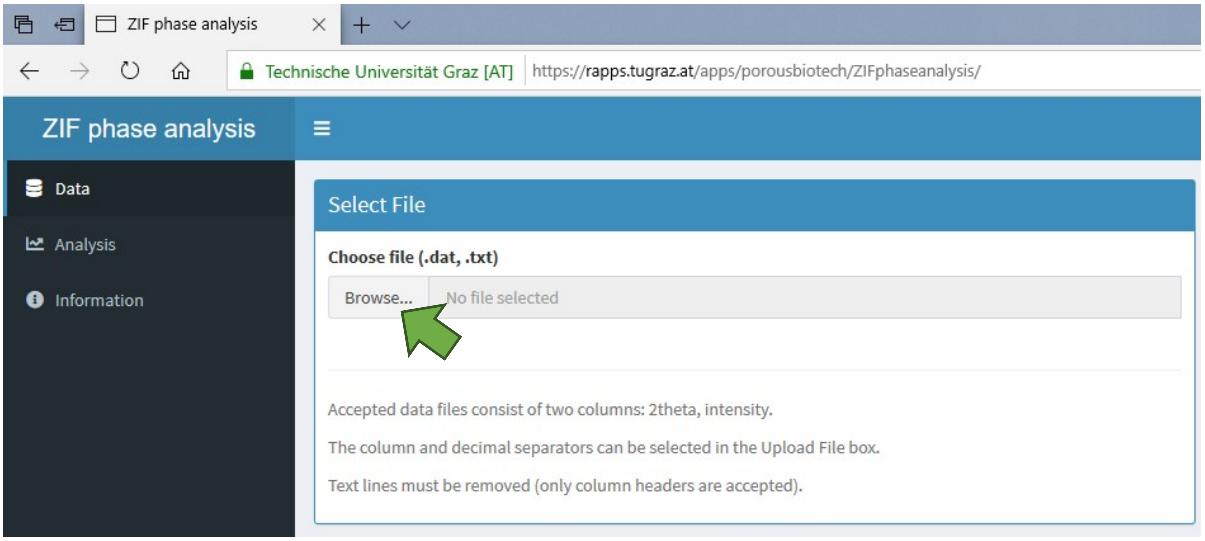

Click "Open" to confirm.

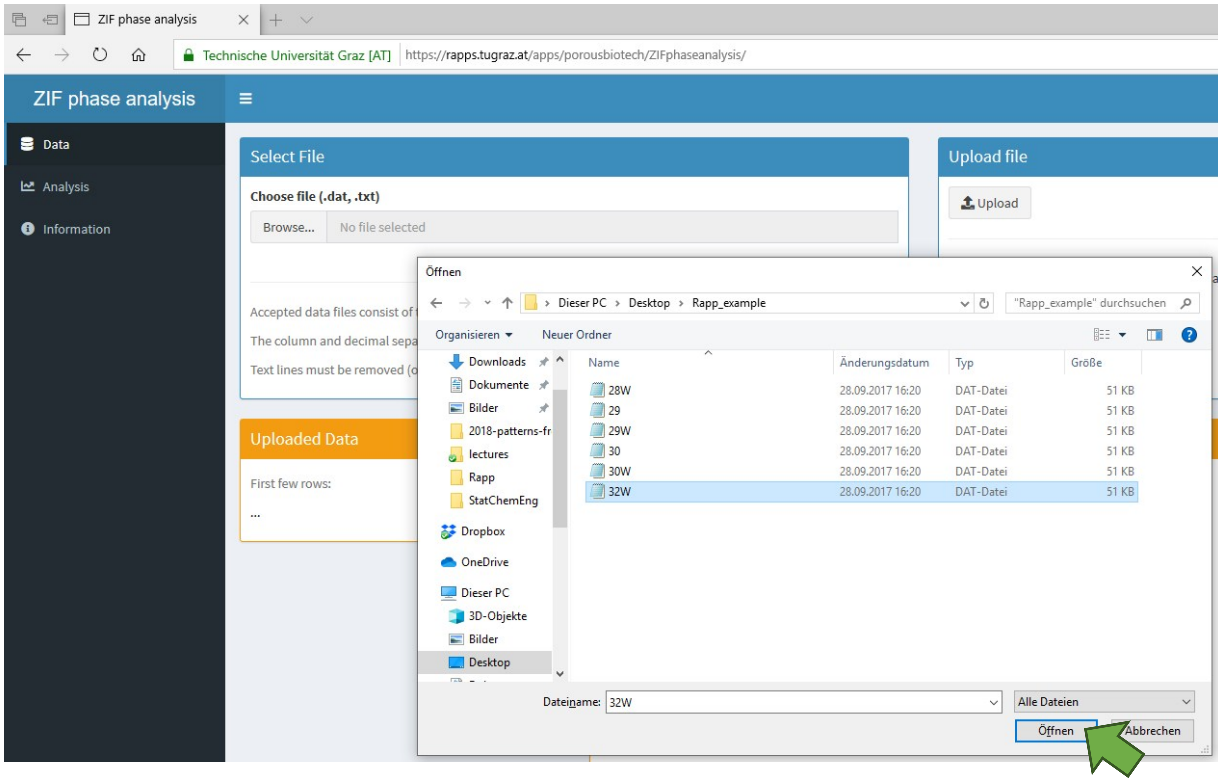

In the "Upload File" box, click on the header check box if column headers are present (default: no header). Then, choose the column separator (default: white space) and the decimal separator (default: point) used in the data file. Finally, click on the "Upload" bottom.

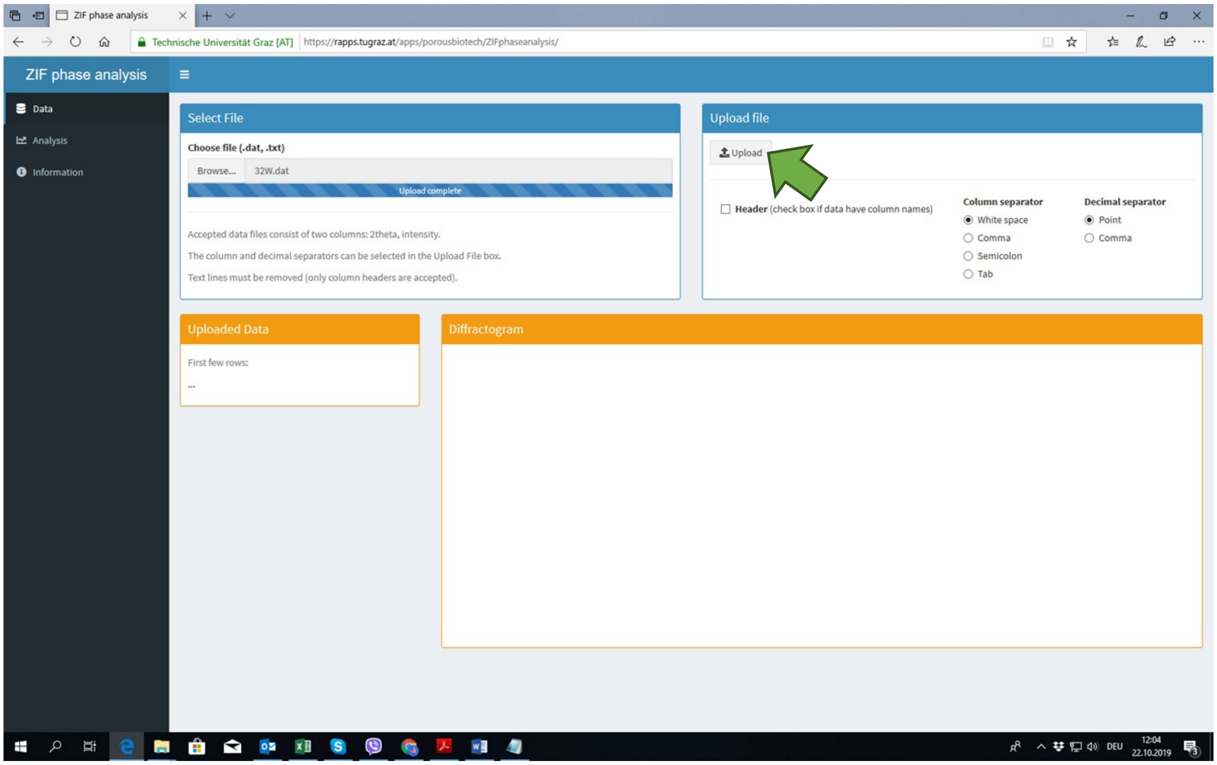

The application runs a series of checks on the file format. In case of problems an error message is returned along with an hint on how to fix the problem. Otherwise, the application visualizes the first few rows of the data file in the “Uploaded Data“ box and the diffraction pattern in the “Diffractogram“ box.

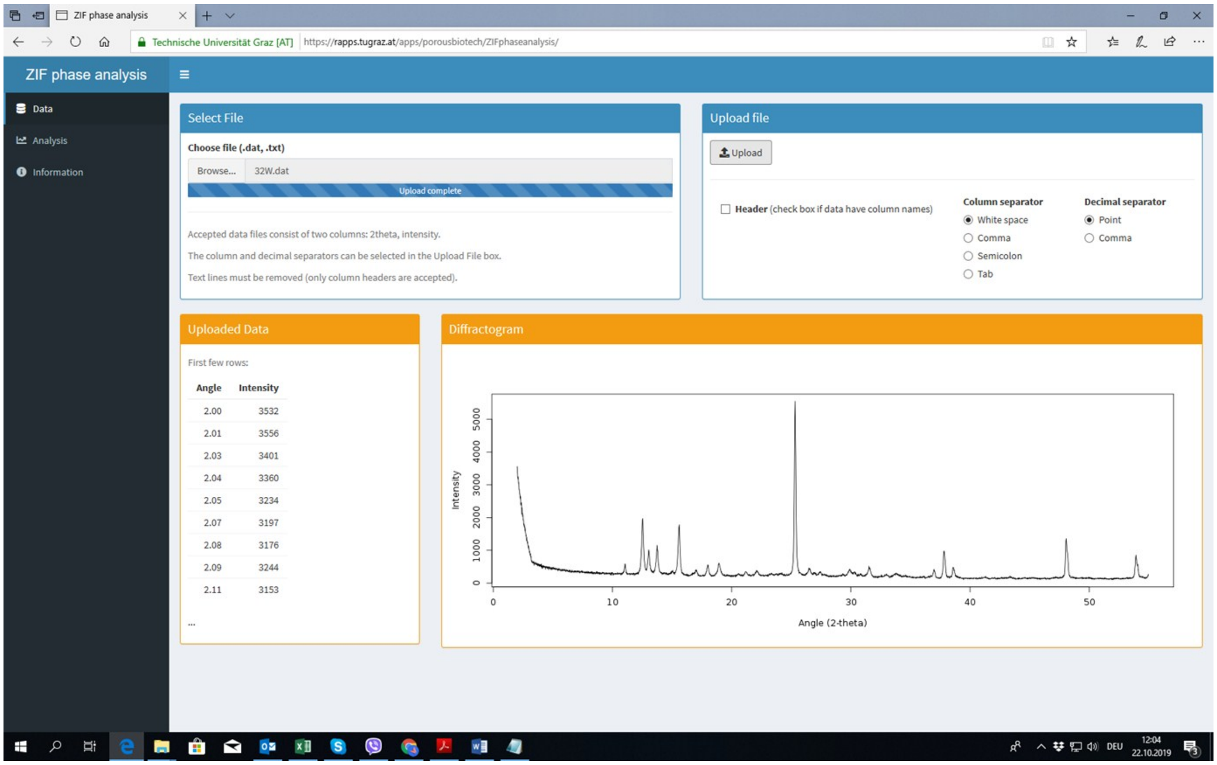

Data file analysis

To visualize the content of the “Analysis“ tab, click on “Analysis“ in the sidebar (top left of screen).

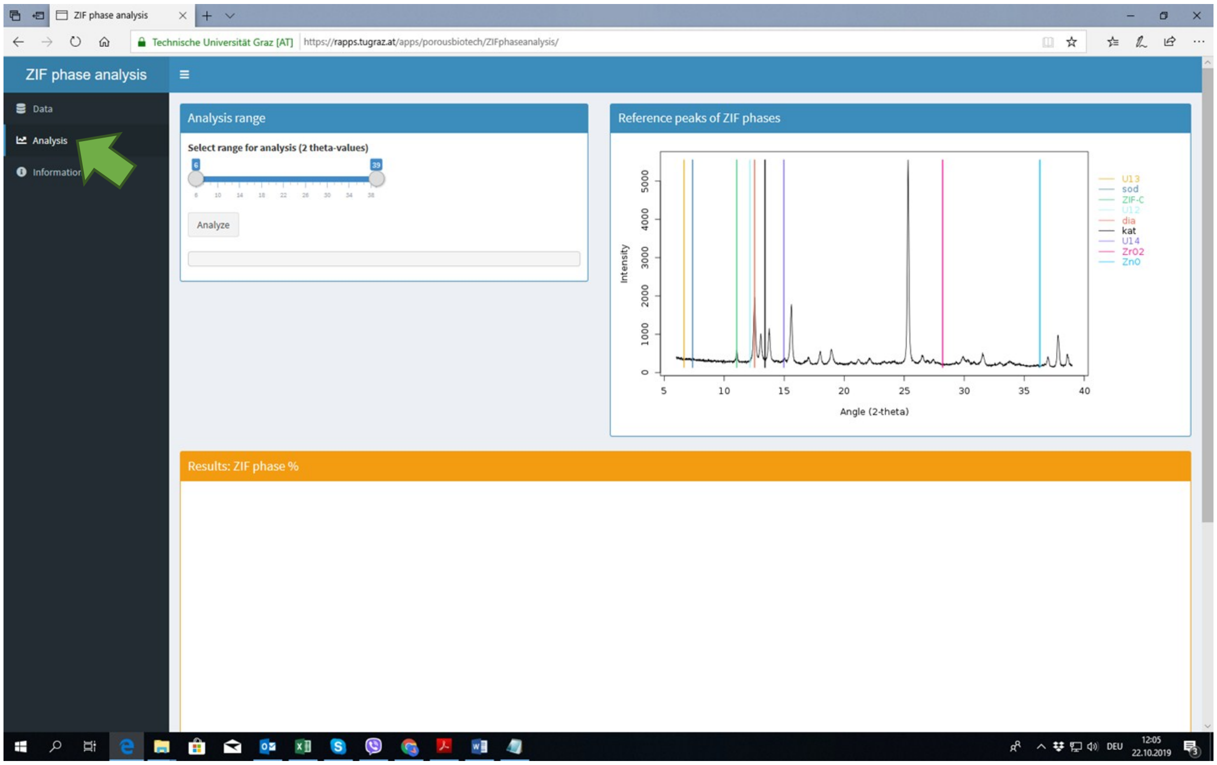

In the “Analysis range“ box it is possible to select the range of 2θ values (default: 6-39) to investigate. The range of 2θ values can be varied by dragging the grey circles in the slidebar.

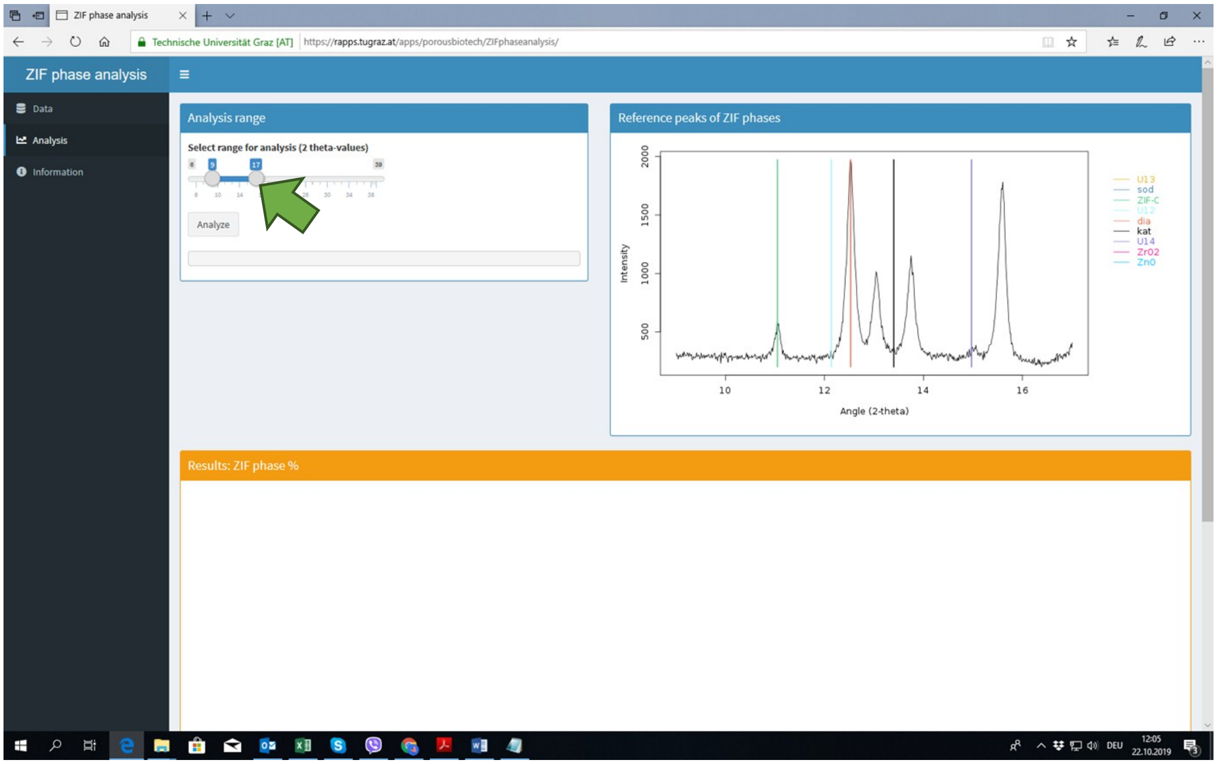

The box “Reference peaks of ZIF phases“ (top right of screen) depicts the diffraction pattern in the desired analysis range. It highlights the reference peaks used for the identification of the ZIF phases, thus providing a preliminary idea of the phase(s) that are present in the analyzed pattern.

To proceed with the analysis, click on the “Analyze“ bottom in the “Analysis range“ box. As the analysis progresses the current step is listed; an alert message appears when the analysis is completed. Details about the implemented statistical analysis are provided in the section “Details of statistical analysis“.

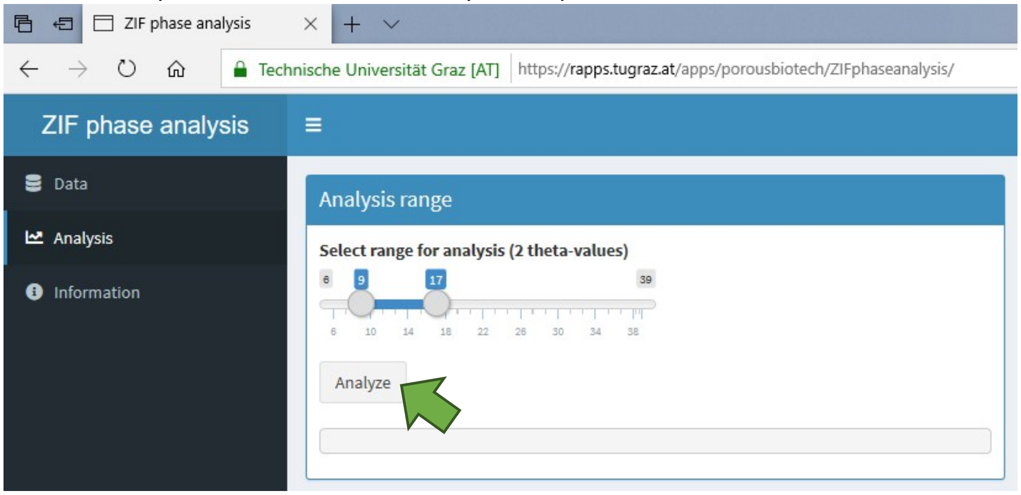

The box “Results: ZIF phase %“ shows the analysis output reporting the identified ZIF phases and their relative intensity. The percentage values of the identified ZIF phases are summarized in a table and visualized in a bar plot.

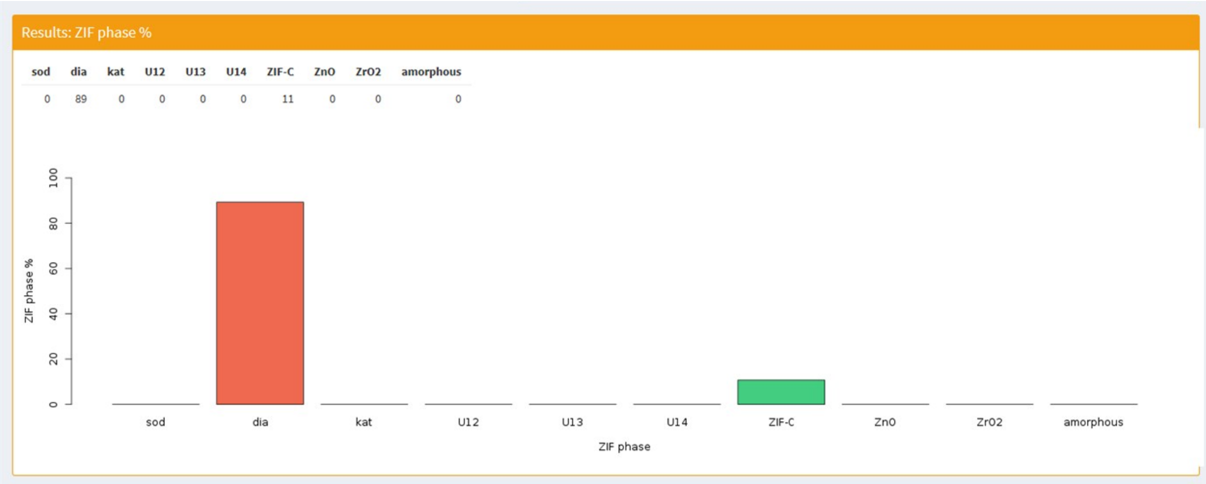

sod	dia	kat	U12	U13	U14	ZIF-C	ZnO	ZrO2	amorphous
0	89	0	0	0	0	11	0	0	0

Finally, a series of collapsed boxes depict the estimates of the peaks associated to the identified ZIF phases. To visualize a specific peak estimate, the user must uncollapse the box by clicking on the "+" sign on the right-hand side of the box.

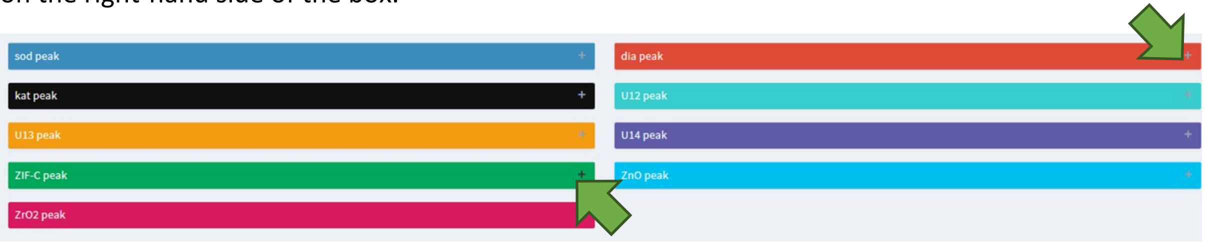

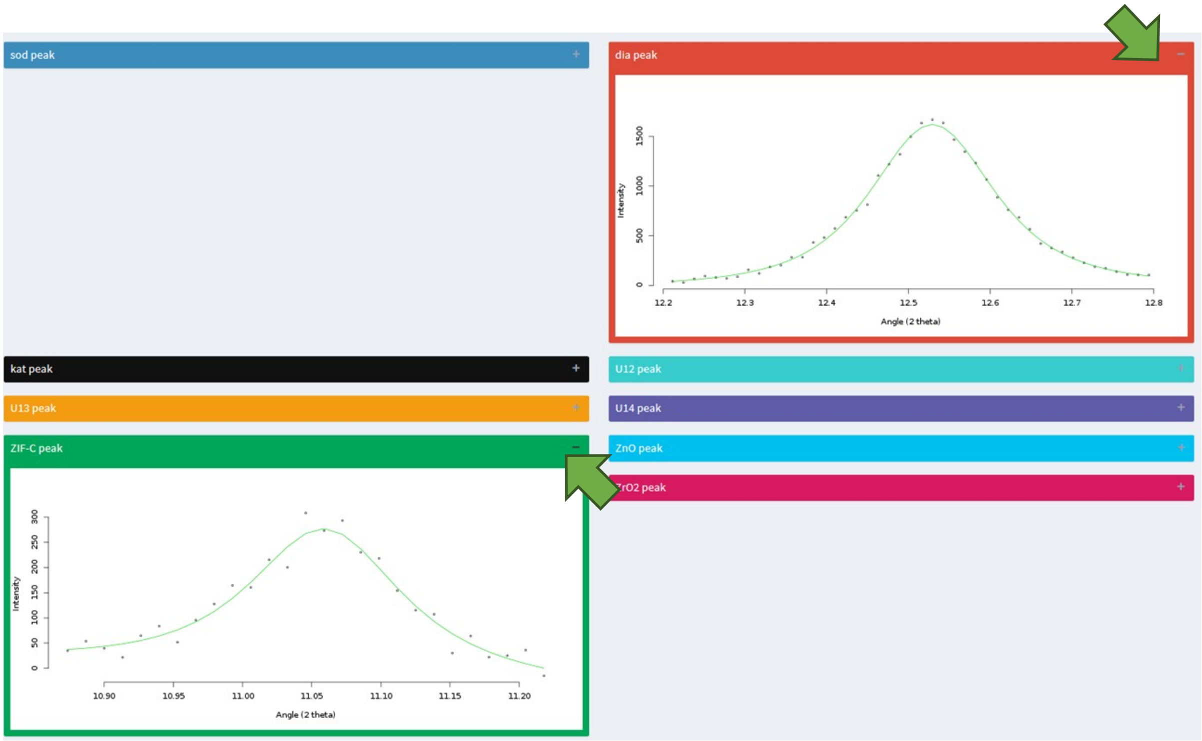

Details of statistical analysis

The statistical analysis of the diffraction pattern is implemented through a customized algorithm consisting of the following main steps:

1. estimate the peaks present in the diffraction pattern;
2. select peaks associated to the ZIF phase(s) of interest;
3. quantify the relative integrated intensity of the identified ZIF phase(s).

1. Peaks estimation

The algorithm makes use of the R package *diffractometry*[viii] to decompose the diffractogram data into baseline, peaks and noise components.[ix] Firstly, the baselinefit funtion is used to identify the approximate peaks positions and the baseline. Secondly, the pkdecomp function is used to obtain accurate estimates of the peaks after baseline substraction.

Both functions depend on a set of parameters. The first set of parameters regulates the desired accuracy of the peaks approximation; these parameters are set to their default values (i.e. tau = 2.5, scl.factor = 2, alpha = 0.1). The second set of parameters regulates the characteristics of the estimated peaks; these paramters are adjusted using information from diffraction patterns of in-house samples. In particular, we set the maximum peak width to maxwdth = 1 as no peak wider than one was observed. We increase the value of the parameter regulating the baseline-peak separation from its default value gam = 1 to gam = 5, as this allows us to identify the peak at 6.65 degrees corresponding to the U13 phase (when using the default value, such a peak was considered to be part of the baseline component). Finally, we reduce the values of the parameters related to the maximum number of attempts to find a good peak approximation (maxiter = 100; default is 10 000) and the number of searched solutions (maxsolutions = 1; default is 3) as this reduces the computational cost of the procedure without affecting the results quality. The output is a list of all the identified peaks (*N*) carrying information about the estimated peaks position and intensity.

2. Peaks selection

Not all the *N* identified peaks are of interest for the current study. The aim of the statistical analysis is to identify if a diffraction pattern presents one or more of the peaks in **Table 1**. The peaks in **Table 1** are expert-selected peaks (reference peaks) referring to five ZIF phases of interest (*sod, dia, kat, U14, ZIF-C),* the *U12* and *U13* phases and two references (ZnO, ZrO2). Each ZIF phase has three to four reference peaks, one of which (highlighted in bold) is more intense than the others.

To identify the relevant peaks, the algorithm compares each of the *N* identified peaks with each peak in **Table 1** using a distance metric between peaks positions. The distance metric is defined by

$$D_{i,p,j} = \min_{i,p,j} |x_i - x_{p,j}^{ref}| \qquad (1)$$

where:

- x_i is the 2θ value for the maximum estimated intensity of peak *i = 1, …, N*;
- $x_{p,j}^{ref}$ is the 2θ value of the *j*th reference peak for phase *p = {sod, dia, kat, U12, U13, U14, ZIF-C, ZnO , ZrO2},* with *j = 1, …, 4*.

If

$$D_{i,p,j} < \tau \tag{2}$$

the *i*th peak is considered to correspond to the *j*th reference peak of phase *p*. Currently, the algorithm uses $\tau = 0.1$, as this provides with the highest correspondence between the ZIF phases identified with the algorithm and the ZIF phases identified using our expert knowledge. Those peaks for which condition (2) holds are selected.

3. ZIF phase quantification

The algorithm further selects only those peaks that correspond to the most intense reference peaks highlighted in bold in **Table 1**. Thus, at most one peak is retained per ZIF phase. We refer to these peaks as shortlisted peaks.

We quantify the magnitude of each shortlisted peak as the area under the peak approximation derived in step *1. Peaks estimation*. To calculate the integral, we use the auc function of the R package MASS[x]. Let A_p be the integral value calculated for the ZIF phase *p* and let RIR_p be the RIR factor[xi] (**Table 1**) for phase *p*. We quantify the relative amount of an identified ZIF phase with the metric defined by

$$RI_p = \frac{(A_p/RIR_p)}{\sum_{k=1}^{9}(A_k/RIR_k)} \tag{3}$$

Thus $0 \le RI_p \le 1$, with $RI_p = 0$ indicating that phase *p* is not present and $RI_p = 1$ indicating that *p* is the only phase present in the investigated sample. Values different from 0 or 1 indicate the presence of multiple phases in the same sample. Instead, if none of the selected phases is identified the algorithms assigns the Amorphous phase.

Phase	reference peaks (2θ, °)				RIR factor
	**1**	**2**	**3**	**4**	
sod	**7,36**	10,45	12,75	18,12	10,67
dia	**12,53**	13,05	13,76	15,57	1,614
kat	12,15	12,25	**13,4**	16,38	2,329
U12	**12,18**	18,43	24,46	24,7	1,614
U13	**6,65**	13,35	20		1,614
U14	9,61	13,98	**14,97**	17,94	1,614
ZIF-C	**11,05**	12,15	13,88	17,87	1,614
ZnO	31,8	34,4	**36,3**		4,875
ZrO2	**28,2**	31,5	34,2		4,7

Table S6. *2ϑ values and RIR factors of selected peaks used for the identification of five ZIF phases, biocomposites with U12 and U13 structure and two references (ZnO and ZrO_2). Each ZIF phase is identified by the presence of three to four selected peaks. The algorithm uses the most intense peak (highlighted in bold) to define if the associated phase is observed in the investigated diffraction pattern. The RIR factors are used in the quantification of the relative peaks integrated intensity. The RIR values for sod (CCDC 963856), dia (CCDC 783838), kat (CCDC 989593), ZnO (COD 9004179) and ZrO_2 (COD 9007485) were obtained from CIF files reported in crystallographic databases (CCDC: Cambridge Crystallographic Data Centre; COD: Crystallographic Open Database). Due to the non-porous nature of U12, U13, U14 and ZIF-C, the RIR factors were assumed to be the same as dia.*

References

[i] W. Liang, R. Ricco, N. K. Maddigan, R. P. Dickinson, H. Xu, Q. Li, C. J. Sumby, S. G. Bell, P. Falcaro and C. J. Doonan, Chem. Mater., 2018, 30, 1069–1077.

[ii] K. Liang, R. Ricco, C. M. Doherty, M. J. Styles, S. Bell, N. Kirby, S. Mudie, D. Haylock, A. J. Hill, C. J. Doonan and P. Falcaro, Nat. Commun., 2015, 6, Article number: 7240

[iii] R Core Team (2019). R: A language and environment for statistical computing. R Foundation for Statistical Computing, Vienna, Austria. URL https://www.R-project.org/

[iv] RStudio Team (2016). RStudio: Integrated Development for R. RStudio, Inc., Boston, MA URL http://www.rstudio.com/

[v] Winston Chang, Joe Cheng, JJ Allaire, Yihui Xie and Jonathan McPherson (2019). shiny: Web Application Framework for R. R package version 1.3.2. https://CRAN.R-project.org/package=shiny

[vi] Winston Chang and Barbara Borges Ribeiro (2018). shinydashboard: Create Dashboards with 'Shiny'. R package version 0.7.1. https://CRAN.R-project.org/package=shinydashboard

[vii] Dean Attali and Tristan Edwards (2018). shinyalert: Easily Create Pretty Popup Messages (Modals) in 'Shiny'. R package version 1.0. https://CRAN.R-project.org/package=shinyalert

[viii] P.L. Davies, U. Gather, M. Meise, D.Mergel, T. Mildenberger. Additional Code by T. Bernholt and T. Hofmeister (2018). diffractometry: Baseline Identification and Peak Decomposition for x-Ray Diffractograms. R package version 0.1-10. https://CRAN.R-project.org/package=diffractometry

[ix] P.L. Davies, U. Gather, M. Meise, D. Mergel, T. Mildenberger (2008): "Residual based localization and quantification of peaks in x-ray diffractograms", Annals of Applied Statistics, Vol. 2, No. 3, 861-886

[x] Venables, W. N. & Ripley, B. D. (2002) Modern Applied Statistics with S. Fourth Edition. Springer, New York. ISBN 0-387-95457-0

[xi] Hubbard, C., & Snyder, R. (1988). RIR - Measurement and Use in Quantitative XRD. *Powder Diffraction, 3*(2), 74-77. doi:10.1017/S0885715600013257
